# Climate change and tree growth in the Khakass-Minusinsk Depression (South Siberia) impacted by large water reservoirs

**DOI:** 10.1038/s41598-021-93745-0

**Published:** 2021-07-12

**Authors:** D. F. Zhirnova, L. V. Belokopytova, D. M. Meko, E. A. Babushkina, E. A. Vaganov

**Affiliations:** 1 Khakass Technical Institute, Siberian Federal University, Abakan, Russia; 2 Laboratory of Tree-Ring Research, University of Arizona, Tucson, USA; 3 Siberian Federal University, Krasnoyarsk, Russia; 4 Sukachev Institute of Forest SB RAS, Krasnoyarsk, Russia

**Keywords:** Climate-change ecology, Forest ecology, Climate change

## Abstract

Regional and local climate change depends on continentality, orography, and human activities. In particular, local climate modification by water reservoirs can reach far from shore and downstream. Among the possible ecological consequences are shifts in plant performance. Tree-ring width of affected trees can potentially be used as proxies for reservoir impact. Correlation analysis and *t*-tests were applied to climatic data and tree-ring chronologies of *Pinus sylvestris* L. and *Larix sibirica* Ledeb. from moisture-deficit habitats in the intermontane Khakass-Minusinsk Depression, to assess modification of climate and tree growth by the Krasnoyarsk and Sayano-Shushenskoe Reservoirs on the Yenisei River. Abrupt significant cooling in May–August and warming in September-March occurred after the launch of the turbines in dams, more pronounced near the Sayano-Shushenskoe dam (up to – 0.5 °C in summer and to + 3.5 °C in winter) than near the Krasnoyarsk Reservoir headwaters (– 0.3 °C and + 1.4 °C). Significant lengthening of the warm season was also found for temperature thresholds 0–8 °C. Shifts of seasonality and intensity occurred in climatic responses of all tree-ring chronologies after development of water reservoirs. Patterns of these shifts, however, depended on species-specific sensitivity to climatic modification, distance from reservoirs, and physiographic regions. Mitigation of climate continentality and extremes by reservoirs appears to have offset possible negative effects of warming on tree growth.

## Introduction

The average rate of global warming over the past 50 years (trend of 0.13 ± 0.03 °C per decade) has turned out to be more than twice the rate previously estimated^1,2^. Warming has been most pronounced in the center of Eurasia, where oceanic influence is minimal^3–5^, but regional trends have varied due to orography affecting atmospheric circulation and climate dynamics^2,6–8^. Past studies show that the dynamics of virtually all climatic parameters can also be significantly modified by anthropogenic factors, such as building of dams^9–13^. Changes associated with dams can have significant environmental consequences on a long timescale^14–16^. Temperature shifts in the impact zone of reservoirs significantly affect terrestrial and aquatic ecosystems, which effects may persist for many kilometers downstream^9,17–19^.

Temperature stratification in a reservoir and the potential range and magnitude of influence on the thermal regime and the environment of the territory adjacent to the reservoir and the downstream river depend strongly on the dimensions of the reservoir^9,10,16,20,21^. It is logical to assume that the magnitude and spatial extent of climate change expand with increasing depth, area and/or length of the reservoir. We expect that this impact can reach many kilometers from the shoreline under favorable orographic conditions, especially in wide intermontane valleys.

Climate change has naturally entailed a shift in the dates of various events in the growth and development of plants. Plant phenology is the simplest and most accessible tool for tracking changes in plant health in response to ongoing climate change^22,23^. In addition, phenology can be considered as a factor in the adaptability and reproductive success of living organisms in natural communities^24,25^. The growing season of plants in temperate and high latitudes is typically limited by temperature thresholds, such that phenological shifts driven by climate warming can significantly alter ecosystem development^26–28^. There is a risk that plants will not be able to adapt fast enough to further rapid shifts in critical temperature thresholds. Warming already led to changes in the timing of various phenophases in tree primary and secondary growth^29–31^. Shifts in tree phenology in response to temperature increase can impact forest productivity and carbon deposition, including annual formation of wood^32^. Both climatic change and its impact on forests can be conveniently appraised with long time series of tree-ring width (TRW).

**Figure 1:**
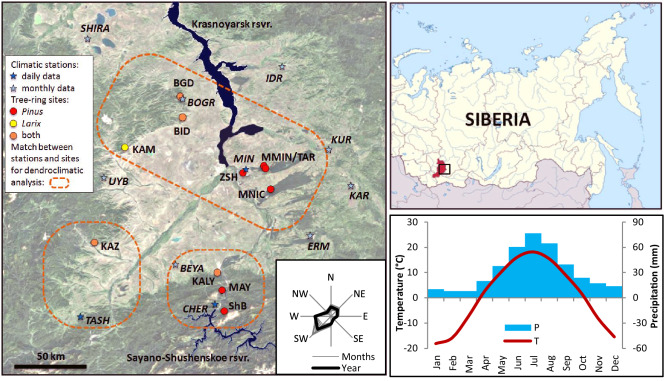
The study area: Khakass-Minusinsk Depression (satellite imagery basemap acquired from ArcGIS Map Wiever, Esri; https://www.arcgis.com/home/webmap/viewer.html). Asterisks represent climatic stations with available daily (dark) and monthly (light) data; circles represent tree-ring sampling sites (red, *Pinus sylvestris* L.; yellow, *Larix sibirica* Ledeb.; orange, both sp.); dash-line areas show areas around climatic stations MIN, TASH, and CHER where their data were used to analyze climatic response of tree-ring chronologies. The inset map shows the location of the study area. Climatic diagram is based on monthly regional data series REG (see “Methods”; 1936–2014). Wind rose shows mean repeatability of wind directions at MIN.

In this study we assessed possible changes in climate and tree-growth dynamics associated with two large reservoirs on the Yenisei River, the world’s largest (annual discharge) Arctic-draining river^33^. The study area, the Khakass-Minusinsk Depression (KhMD), is a large intermountain valley with a strongly continental climate in southern Siberia, at the northern edge of the vast Altai-Sayan mountainous region (Fig. 1). The KhMD is bounded by the Kuznetsk Alatau in the west, by the Western Sayan in the south, and by the Eastern Sayan in the east. The continental location in combination with the rain shadow of the surrounding mountains reduces the moisture available from oceanic air masses. The average annual precipitation (P) is 250–500 mm, and vegetation is mainly steppe and forest-steppe. The climate is characterized by large seasonal and daily air temperature (T) variation. Summers are hot and winters are frosty with little snow. The well-defined orographic boundaries of the basin provide commonality and synchronization of climatic fluctuations, such that we can speak of a distinctive KhMD regional climate.

The KhMD is crossed south-to-north by the Yenisei, on which two massive reservoirs were created in the second half of the twentieth century. During the construction of the dam of the Krasnoyarsk Reservoir (water volume of 73.3 billion m^3^, area of 2000 km^2^, and depth of up to 105 m), the Yenisei was blocked in 1967. The first turbines of the power plant were launched the next winter, and by 1970 the reservoir reached the designed water level. Water backed upstream from the dam to the cities of Abakan and Minusinsk, located in the middle of the KhMD, so that the headwaters of the Krasnoyarsk Reservoir cross most of the valley. The deeper and narrower Sayano-Shushensky Reservoir (volume of 31.3 billion m^3^, area of 621 km^2^, and depth of up to 220 m) in the Western Sayan Mountains filled over a longer interval, 1975–1990. The turbines were launched during 1980–1985, resulting in alteration of Yenisei water temperatures downstream in the southern part of the KhMD.

Enough time has passed since building of the dams for the establishment of a new stable climatic regime, and sufficiently long climate series at several stations are available for comparison of conditions before and after the start of possible influence by the reservoirs. Two dominant conifer species in the area, Scots pine (*Pinus sylvestris* L.) and Siberian larch (*Larix sibirica* Ledeb.), were selected as indicators of the influence of regional warming and reservoirs on the regional forest ecosystems. In this paper we applied a network of tree-ring chronologies developed between 2012 and 2019 in combination with monthly and daily P and T station climate records (Table 1) in a detailed spatial–temporal analysis of the regional dynamics of the main climatic factors and characteristics of the vegetative season, and assessed possible impact of reservoirs on climate and tree growth.

**Table 1:** Sampling sites and climatic stations.

Site/station		Coordinates			Slope	Description	Sampled species
Name	Code	N	E	h, m asl			
Zelyony Shum	ZSH	53.65	91.60	300	Flat	Birch-pine, steppe zone	PS
Malaya Minusa	MMIN	53.75	91.77	300	Flat		PS
Taraska	TAR	53.75	91.77	350	South		PS
Malaya Nichka	MNIC	53.62	92.05	380	Flat	Aspen-birch-pine, steppe zone	PS
Bograd	BGD	54.20	90.83	600	South	Birch-larch-pine, forest-steppe	PS LS
Bidja	BID	54.00	91.02	660	South		PS LS
Kamyzyak	KAZ	53.92	90.60	685	South		PS LS
Kazanovka	KAM	53.22	90.08	730	South	Birch-larch, forest-steppe	LS
Kaly	KALY	53.05	91.28	520	South	Larch-pine, 10 km from steppe	PS LS
Mayna	MAY	52.97	91.35	400	South	Pine, forest-steppe	PS
Shushensky Bor	SHB	52.83	91.45	520	South	Birch-larch-pine, sub-taiga	PS
Minusinsk	MIN	53.72	91.10	255	Climatic stations		
Tashtyp	TASH	52.80	89.88	455			
Cheryomushki	CHER	52.83	91.38	330			
Shira	SHIRA	54.50	59.93	475			
Bograd	BOGR	54.23	90.83	430			
Uybat	UYB	53.72	90.37	525			
Beya	BEYA	53.05	90.92	468			
Idrinskoe	IDR	54.37	92.13	282			
Kuragino	KUR	53.9	92.68	288			
Karatuzskoe	KAR	53.6	92.85	320			
Ermakovskoe	ERM	53.3	92.42	298			

Sampled tree species encoded as PS—*Pinus sylvestris* L., LS—*Larix sibirica* Ledeb.

## Results

### Spatio-temporal climatic patterns

Long-term warming trends in the study region are at least twice the rate of global warming, especially in winter (Supplementary Fig. S1). This can be seen in T records for MIN and CHER, climatic stations representing conditions nearest the reservoirs (Fig. 1), and in the KhMD regional series (REG), an average for stations located near boundaries of the valley. In November–March, T increased over 1936–2014 by 0.14 °C per decade in Northern Hemisphere, by 0.36 °C on regional scale (REG), and by 0.57 °C at MIN. The warming rate over 1951–2014 at CHER was even steeper: 0.92 °C per decade in comparison with regional warming rate of 0.38 °C and global one of 0.19 °C per decade for this period. In contrast, during the warm season of April–October warming rates were comparable at all three spatial scales.

Temporal shifts in monthly mean temperature (ΔT) relative to the regional averages were recorded at stations MIN and CHER after launching of the turbines at the dams (Table 2). At MIN, the relative warming extends from October to March and reaches a maximum of ΔT = 1.37 °C in January. Summer cooling at MIN (June–August) has a range of 0.2–0.33 °C. The relative warming at CHER (September–March) reaches a maximum of ΔT = 3.52 °C in December; summer cooling (May–July) reaches a maximum of ΔT = − 0.51 °C in June. Shifts in P at MIN and CHER relative to the regional average are less significant that the shifts in T. A shift toward more P in summer is suggested at both MIN and CHER. Time series plots show that the difference between station and regional monthly T (*d*T) was changing at MIN and CHER during the periods of filling the respective reservoirs—3 years for Krasnoyarsk, 15 years for Sayano-Shushenskoe (Supplementary Fig. S2). The most abrupt changes, confirmed with a *t*-test, occurred immediately after launch of the turbines at the dams.

**Table 2:** Impact of reservoirs on difference of local and regional climate.

Months	MIN						CHER					
	*d*T = T_MIN_–T_REG_ (°C)			*d*P = P_MIN_–P_REG_ (mm)			*d*T = T_CHER_–T_REG_ (°C)			*d*P = P_CHER_–P_REG_ (mm)		
	1936–1967	1968–2014	ΔT	1936–1967	1968–2014	ΔP	1951–1979	1980–2014	ΔT	1951–1979	1980–2014	ΔP
Jan	− 2.10 ± 1.38	− 0.73 ± 0.81	+ 1.37*	− 2 ± 3	− 2 ± 4	0	3.10 ± 0.88	6.45 ± 1.19	+ 3.35*	0 ± 6	1 ± 4	+ 1
Feb	− 1.79 ± 1.53	− 0.65 ± 0.88	+ 1.14*	− 1 ± 4	− 1 ± 3	0	3.23 ± 0.90	5.98 ± 0.95	+ 2.75*	3 ± 6	1 ± 3	− 2
Mar	− 0.31 ± 1.41	0.64 ± 0.65	+ 0.95*	1 ± 5	− 2 ± 4	− 3	2.56 ± 0.64	4.12 ± 0.82	+ 1.56*	5 ± 6	3 ± 6	− 2
Apr	1.13 ± 0.58	1.14 ± 0.33	+ 0.01	− 5 ± 7	− 4 ± 8	+ 1	1.49 ± 0.45	1.72 ± 0.47	+ 0.23	11 ± 14	12 ± 13	+ 1
May	1.31 ± 0.34	1.20 ± 0.24	− 0.11	− 4 ± 11	− 4 ± 10	0	1.11 ± 0.48	0.71 ± 0.38	− 0.40*	22 ± 20	24 ± 26	+ 2
Jun	1.67 ± 0.44	1.47 ± 0.24	− 0.20*	− 9 ± 14	− 2 ± 18	+ 7*	0.66 ± 0.50	0.15 ± 0.67	− 0.51*	16 ± 23	25 ± 29	+ 9
Jul	1.88 ± 0.34	1.63 ± 0.31	− 0.25*	− 15 ± 22	− 5 ± 24	+ 10	0.60 ± 0.33	0.33 ± 0.39	− 0.27*	21 ± 29	29 ± 31	+ 8
Aug	1.78 ± 0.31	1.45 ± 0.25	− 0.33*	− 13 ± 20	− 2 ± 25	+ 11*	0.96 ± 0.23	1.01 ± 0.30	+ 0.05	29 ± 25	23 ± 25	− 6
Sep	0.98 ± 0.28	0.95 ± 0.31	–0.03	1 ± 14	3 ± 12	+ 2	1.15 ± 0.25	1.85 ± 0.29	+ 0.70*	17 ± 15	13 ± 17	–4
Oct	0.37 ± 0.58	0.62 ± 0.38	+ 0.25*	− 5 ± 10	1 ± 8	+ 6*	1.74 ± 0.35	2.96 ± 0.29	+ 1.22*	7 ± 14	18 ± 17	+ 11*
Nov	− 0.21 ± 1.01	0.44 ± 0.64	+ 0.65*	− 3 ± 7	− 3 ± 6	0	2.66 ± 0.66	4.77 ± 0.89	+ 2.11*	4 ± 8	8 ± 15	+ 4
Dec	− 1.24 ± 1.41	− 0.24 ± 0.98	+ 1.00*	− 1 ± 7	− 3 ± 5	– 2*	3.03 ± 0.93	6.55 ± 1.23	+ 3.52*	2 ± 7	4 ± 6	+ 2

For each monthly climate variable and station (Minusinsk—MIN, Cheryomushki—CHER), the first two columns show mean ± standard deviation of the difference *d* between station and regional (REG; see text) values over sub-periods before and after launching of the turbines in the respective dams; the third column (Δ) indicates the change in that difference from the earlier to later sub-period, significant (*p* < 0.05) changes are written in bold.

The shifts reported above occurred against a background of regional climate change. The regional series itself shows a pattern of warming in every month of the year pre-dam to post-dam for both reservoirs (Supplementary Fig. S3). The regional warming is stronger in the cold season, a contrast magnified near the reservoirs, especially at CHER.

Reservoirs had a significant impact on derivative temperature variables summarizing heat characteristics of the vegetative season (Table 3, Supplementary Fig. S4). The Krasnoyarsk Reservoir is associated with an earlier start to the season by a significant 6 days for threshold temperatures T_thr_ = 0 °C and 5 °C at nearby station MIN and by 5 days at the most remote station TASH (Fig. 1). Corresponding changes in the end date of the vegetative season were insignificant at both stations. The duration of the growing season and the number of degree-days in the season increased at both MIN and TASH for all T_thr_. The increase in degree-days was significant at TASH for T_thr_ = 0–5 °C. The shifts in vegetative season were much more pronounced for the Sayano-Shushenskoe Reservoir. Changes were most significant at CHER, which is only 5 km from the dam. After launching of the turbines, spring started up to 7 days earlier, and autumn started up to 9 days later. The duration of the vegetative season increased by as much as 15 days, and the number of degree-days increased significantly for the lower T_thr_. Changes at TASH were smaller than at CHER, but were significant at the lowest threshold T_thr_ = 0 °C for all four characteristics, with spring starting 5 days earlier and autumn ending 5 days later.

**Table 3:** Impact of the reservoirs on temperature variables.

Temperature threshold, T_thr_	Krasnoyarsk reservoir						Sayano-Shushenskoe reservoir					
	Near: MIN			Far: TASH			Near: CHER			Far: TASH		
	1936–1967	1968–2014	∆	1936–1967	1968–2014	∆	1951–1979	1980–2014	∆	1951–1979	1980–2014	∆
**Date of stable temperature crossing T**_**thr**_** in spring (days)**												
0 °C	5 Apr ± 9	30 Mar ± 9	− 6*	8 Apr ± 9	3 Apr ± 9	− 5*	1 Apr ± 9	25 Mar ± 11	− 7*	7 Apr ± 8	2 Apr ± 9	− 5*
5 °C	16 Apr ± 10	10 Apr ± 9	− 6*	17 Apr ± 8	14 Apr ± 10	− 3	11 Apr ± 8 /)	6 Apr ± 10	− 5*	17 Apr ± 8	13 Apr ± 10	− 4
10 °C	3 May ± 8	3 May ± 11	0	10 May ± 9	10 May ± 11	0	8 May ± 11	5 May ± 12	− 3	11 May ± 9	9 May ± 11	− 2
**Date of stable temperature crossing T**_**thr**_** in autumn (days)**												
0 °C	25 Oct ± 8	26 Oct ± 8	+ 1	22 Oct ± 8	24 Oct ± 9	+ 2	28 Oct ± 7	6 Nov ± 10	+ 9*	21 Oct ± 9	26 Oct ± 9	+ 5*
5 °C	11 Oct ± 9	9 Oct ± 10	− 2	6 Oct ± 9	6 Oct ± 8	0	16 Oct ± 9	21 Oct ± 9	+ 5*	7 Oct ± 8	7 Oct ± 9	0
10 °C	6 Sep ± 9	9 Sep ± 8	+ 3	31 Aug ± 9	1 Sep ± 7	+ 1	9 Sep ± 9	12 Sep ± 8	+ 3*	1 Sep ± 8	3 Sep ± 6	+ 2
**Duration of season with temperature above T**_**thr**_** (days)**												
0 °C	202 ± 10	210 ± 11	+ 8*	197 ± 10	204 ± 13	+ 7	211 ± 10	226 ± 15	+ 15*	197 ± 11	207 ± 12	+ 10*
5 °C	178 ± 14	182 ± 14	+ 4	172 ± 11	176 ± 13	+ 4	188 ± 12	198 ± 15	+ 10*	173 ± 10	177 ± 14	+ 4
10 °C	113 ± 12	116 ± 14	+ 3	102 ± 15	104 ± 12	+ 2	115 ± 10	119 ± 15	+ 4*	103 ± 13	106 ± 12	+ 3
**Sum of active temperatures above T**_**thr**_** (°C·days)**												
0 °C	2525 ± 144	2586 ± 135	+ 61	2213 ± 123	2287 ± 133	+ 74*	2473 ± 104	2644 ± 138	+ 172*	2205 ± 116	2321 ± 127	+ 116*
5 °C	1603 ± 132	1631 ± 109	+ 28	1317 ± 112	1367 ± 106	+ 50*	1521 ± 97	1601 ± 114	+ 80*	1318 ± 101	1389 ± 106	+ 71*
10 °C	875 ± 106	886 ± 93	+ 11	636 ± 84	666 ± 80	+ 30	2473 ± 104	2644 ± 138	+ 172*	2205 ± 116	2321 ± 127	+ 116*

For each reservoir and station (Minusinsk—MIN, Cheryomushki—CHER, Tashtyp—TAH), the first two columns show *mean* ± *standard deviation* over sub-periods before to after launching of the turbines; the third column (Δ) shows difference in means between sub-periods, significant (p < 0.05) differences are written in bold.

**Figure 2:**
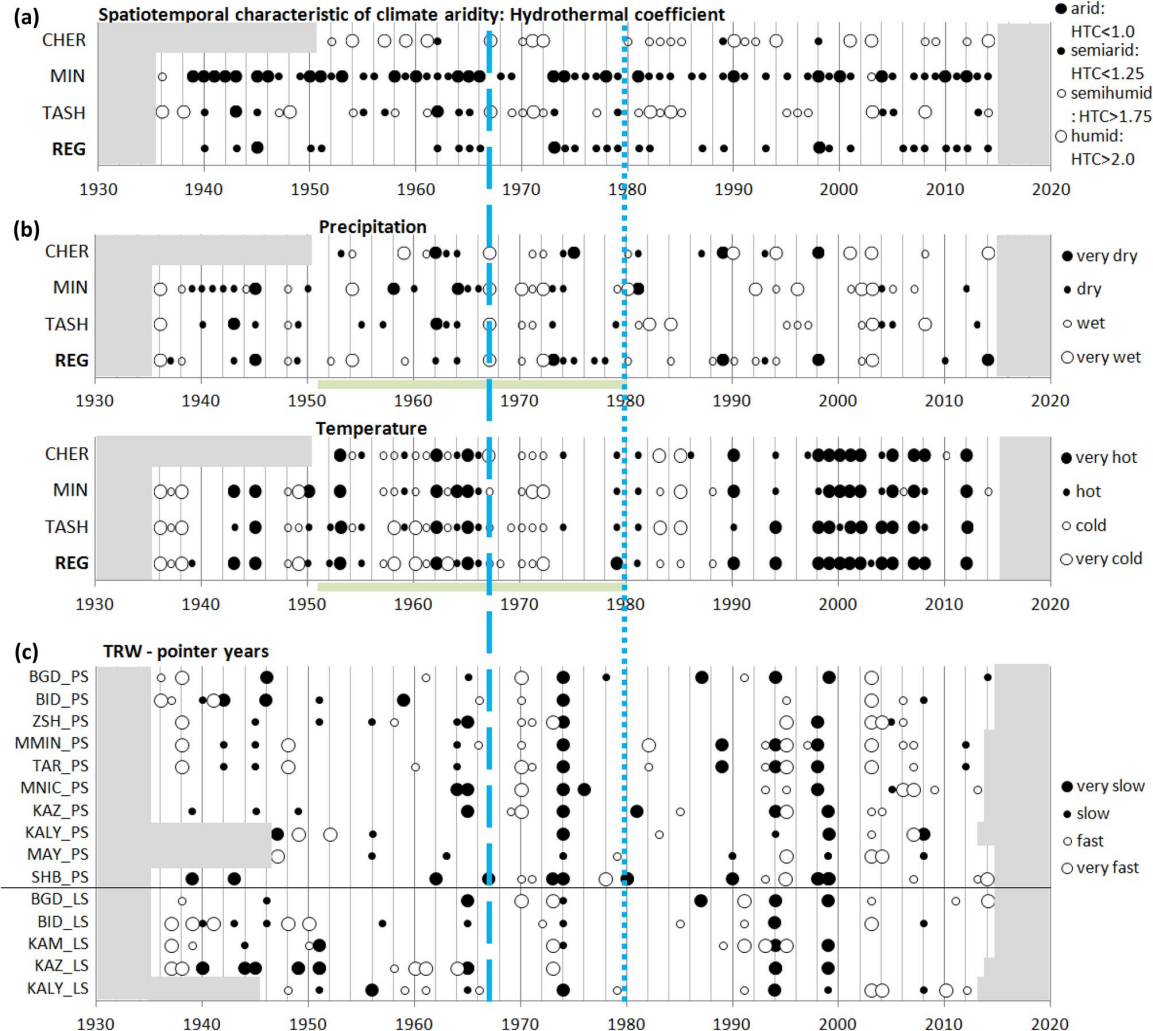
Dynamics of extremes in climatic variables and tree growth. (**a**) Climate aridity expressed as Selyaninov Hydrothermal coefficient HTC for May–September; (**b**) Extreme cold/hot and dry/wet years for May–September temperature T and precipitation P; (**c**) Pointer years indicated by extremes in TRW chronologies. Vertical lines mark launch of the turbines in dams of Krasnoyarsk Reservoir (dashed) and Sayano-Shushenskoe Reservoir (dotted).

The frequency of climate events, defined by specific probability points in the tails of distributions (see “Methods”), also changed with launching of the turbines at the dams. The dynamics of climate events for the region (REG) and stations CHER, MIN and TASH are summarized in time series plots of extremes in Selyaninov hydrothermal coefficient (HTC; see “Methods”), P, and T for May–September (Fig. 2a,b). This interval corresponds roughly to the vegetative season and to the period with mean daily T above 10 °C (see Table 3). Aridity as measured by HTC is greatest in the center of the valley (MIN), and decreases towards the foothills (REG and TASH). Station CHER, at the edge of the Western Sayan Mountains, receives more precipitation, and its climate is classified as humid in many years. Neither regional warming of recent decades nor creation of reservoirs impacted aridity. A decrease in frequency of dry events and increase in frequency of wet events was observed at MIN and TASH after the launch of the second dam. The event series for T exhibits pronounced changes at the decadal time scale, including a decrease in hot events and corresponding increase in cool events after launching of the turbines at both dams. From 1998 to 2008, a period of high T was observed throughout the basin against a background of normal or increased P and no significant change in aridity.

### TRW chronologies and their climatic response

The TRW site chronologies (Fig. 1, Table 1), based on 13–144 core samples per site, range in length from 66 to 316 years (Supplementary Table S1). Mean ring width, which can be expected to vary depending on tree age and habitat, ranges from 0.94 to 2.85 mm with no systematic difference for larch and pine. Variability of the chronologies, as measured by the standard deviation and mean sensitivity, is somewhat higher in the foothills of the Kuznetsk Alatau than elsewhere, and reaches a maximum in both species at the KAZ site. The strength of the common signal (*r-bar*) ranges from 0.31 to 0.56, and chronologies are representative of the population tree-growth variations (EPS > 0.85) for the entire period of climatic analysis at all sites except the youngest forest stands at KALY and MAY.

The correlation matrix of TRW chronologies is positive for all chronology pairs, but correlations vary widely depending on species and geographical proximity (Supplementary Table S2). Correlations range from *r* = 0.09 for different species from distant regions (SHB_PS & BID_LS) to *r* = 0.95 for pines from adjacent sites (MIN_PS and TAR_PS). Correlations are especially high for the same species and region: for example, *r* = 0.63–0.95 for pine within the Minusinsky Bor, and *r* = 0.63–0.73 for larch in the northern part of the Kuznetsk Alatau foothills. Correlations are also high between species within the same site (*r* = 0.55–0.73).

Growth of both species is correlated positively with P and negatively with T for all considered chronologies. Analysis of TRW correlations with monthly T and P (Supplementary Table S3) showed that pine chronologies from Minusinsky Bor and northern part of Kuznetsky Alatau foothills (BGD, BID) are characterized by a weak T influence at the end of the previous season (the strongest correlations are *r* = − 0.17 to − 0.32) and in the current May, and by a maximum T influence in summer (*r* = − 0.24 to − 0.36). Influence of P is significant in previous August–September (*r* = 0.32 to 0.46) and November (*r* = 0.33 to 0.49), and current May–July (*r* = 0.25 to 0.46). For all other chronologies from the Kuznetsk Alatau, significant T impact is observed in the previous August–September (*r* = − 0.22 to − 0.38) and the current May–June (*r* = − 0.23 to − 0.38). The reaction to P is shifted to previous July–August (*r* = 0.23 to 0.44) and current May–June (*r* = 0.26 to 0.42). The chronologies of the Western Sayan foothills have a significant response to T and P in previous August (*r* = − 0.31 to − 0.47 and *r* = 0.23 to 0.44, respectively) and current May (*r* = − 0.29 to − 0.35 and *r* = 0.30 to 0.43); an effect of P was also noted in January-March (*r* = 0.22 to 0.33).

**Figure 3:**
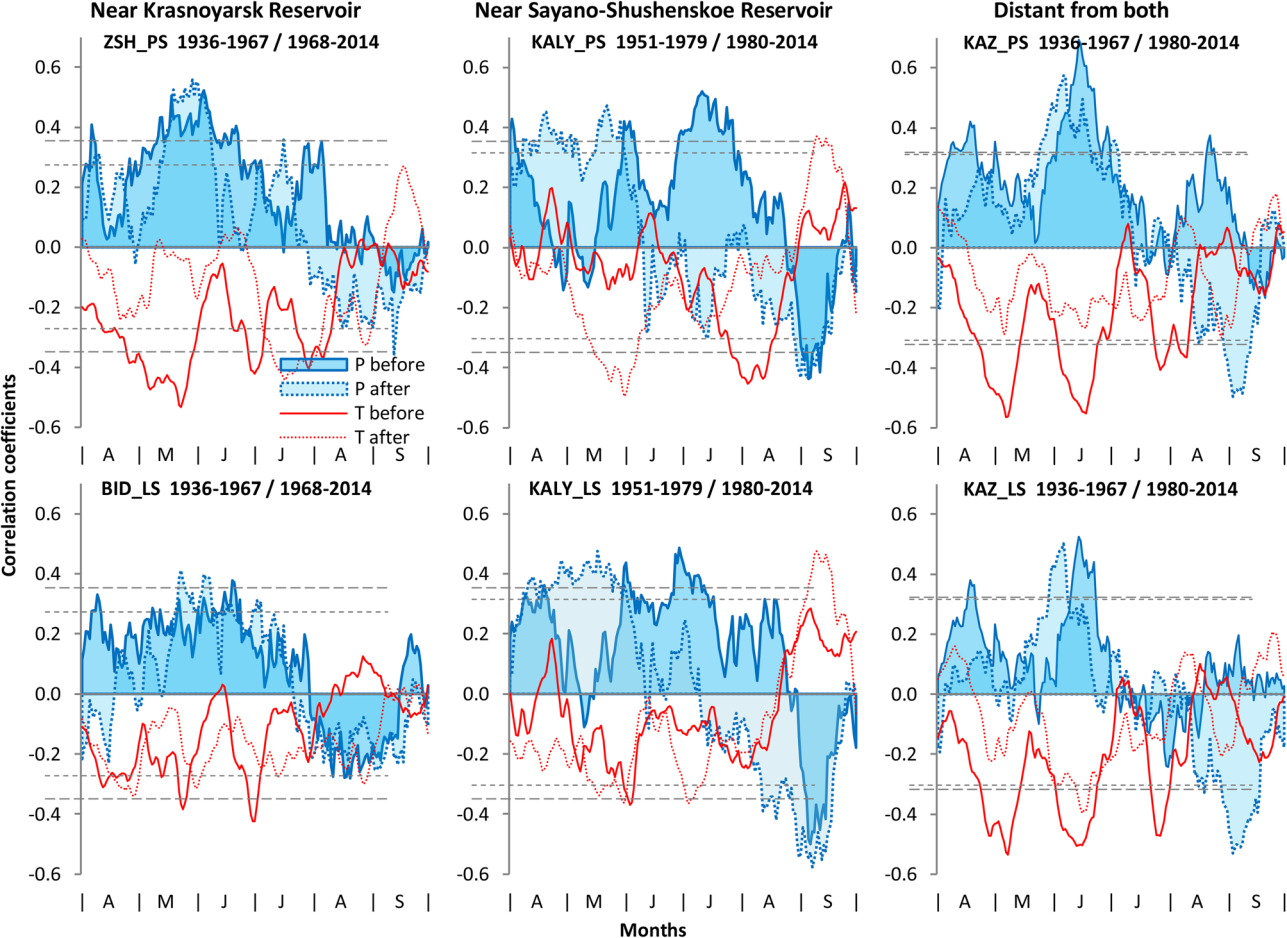
Windowed (21-day) correlations of TRW chronologies with temperature T and precipitation P during vegetative season over sub-periods before and after launching of the turbines in dams on example of 6 chronologies. Correlations plotted at center of 21-day window. Dashed horizontal lines represent significance at *p* < 0.05 for the first (long dashes) and the second (short dashes) sub-periods.

Windowed correlations of daily P and T with TRW chronologies show consistent patterns of change in the relationship of climate to tree-growth after launching of the turbines at dams (Fig. 3; Supplementary Fig. S5). Near the Krasnoyarsk Reservoir, the positive P response strengthens from mid-May to mid-June, and weakens in other periods. The typically negative T response weakens, especially in April–May. These shifts are more pronounced for pine, where the season of highest correlation shifts to earlier dates for P and later dates for T. Similar but less pronounced changes occur farther from the reservoir. Even sharper changes in climate response occur at sites close to the Sayano-Shushensky Reservoir: a previously weak response to P from early April to mid-June strengthened greatly, but from mid-June a positive P response changed to negative. The most obvious shift in the negative T response is the appearance of correlation peaks from late May to the first half of July, but these peaks do not always reach significance.

Numerous “pointer years” of TRW, i.e., years with TRW more extreme than specified probability points of its distribution (see “Methods”), were synchronous over the study area (Fig. 2c). Extreme growth departures were synchronous notably not only within each species, but to a lesser extent between species. The plots show a clear association of pointer years with unusual P and T departures in the vegetative season. Wide annual rings coincide with a cool or wet vegetative season in 1937–1938, 1948, 1970, 2003. Conversely suppression of growth in 1944–1945, 1965, 1974, 1994, 1998–1999 coincide with drought or high T in the current or previous year’s warm season.

**Figure 4:**
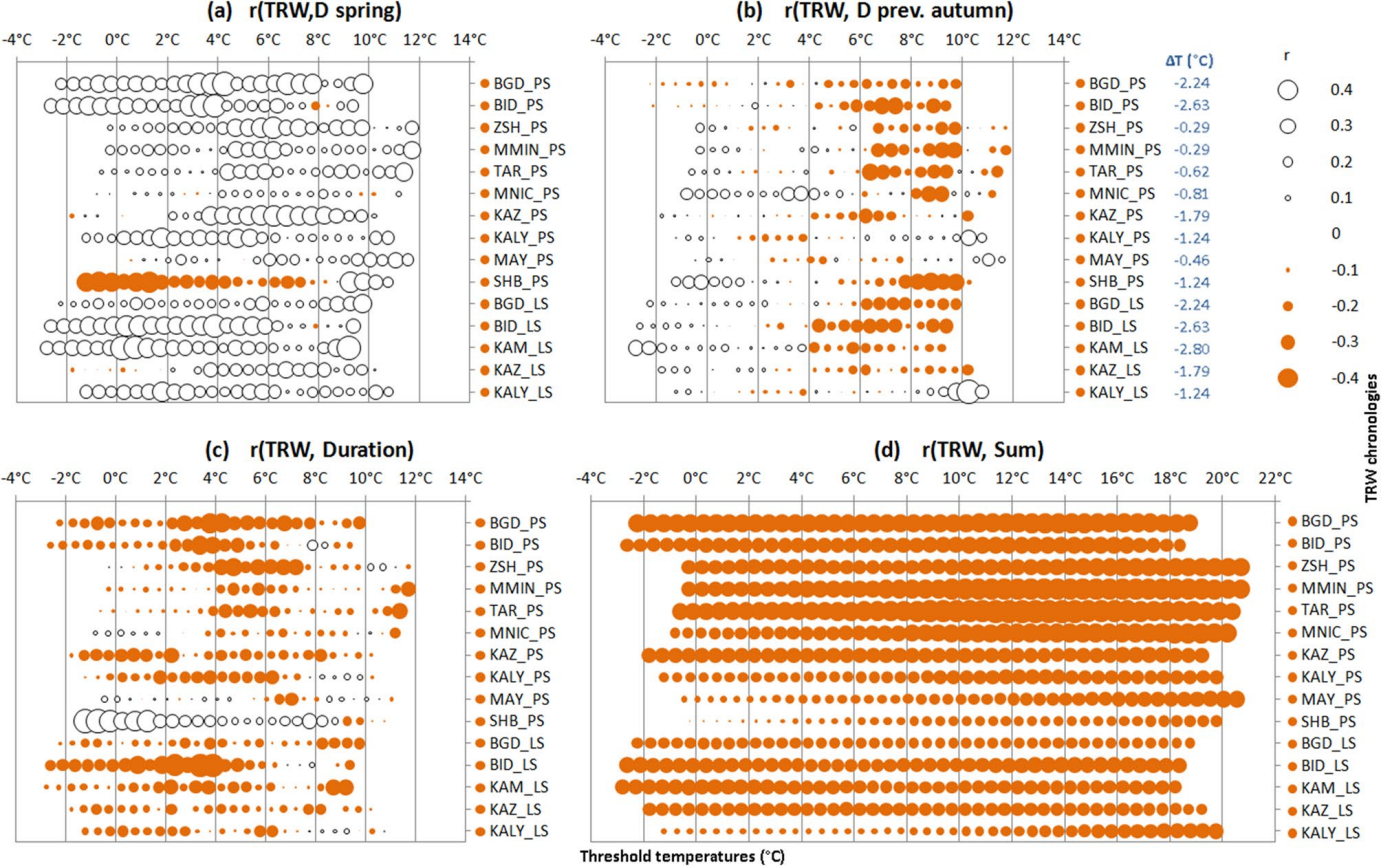
Correlations of TRW chronologies with climatic variables derived from station daily air temperature. (**a**) Date of T crossing threshold temperature values T_thr_ in spring (D spring). (**b**) Date of T crossing T_thr_ in autumn of previous year (D prev. sutumn). (**c**) Duration of period between T crossing T_thr_ in spring and autumn. (**d**) Cumulative temperature sum (degree-days) above T_thr_. Temperature at sampling sites was corrected from temperature at climate stations as described in “Methods”, temperature correction values are presented near legend (blue).

The sensitivity of tree growth to climate variations is dependent on tree phenology and the timing of the growth season as delineated by crossing of T_thr_. All chronologies except pine in the sub-taiga zone correlate positively with the dates of specified T_thr_ in spring (Fig. 4a): the later T rises above T_thr_ = − 3–12 °C (considering the temperature differences at the stations and sampling sites), the greater the growth of trees. For both species this relationship is more pronounced in the northern part of the Kuznetsk Alatau than elsewhere. At the ShB site, on the contrary, pine growth is facilitated by the earlier onset of positive T. The highest correlations were recorded for T_thr_ = 3–8 °C for pine and T_thr_ = 0–6 °C for larch. As for the dates of the reverse temperature transition in autumn, the correlations of TRW and the transition dates in the year of growth are insignificant at *p* < 0.05 (not shown). Correlations with the autumn temperature transition dates of the previous year, however, are negative for pine at BGD, MMIN, TAR, MNIC, SHB and for larch at BGD and BID in the range T_thr_ = 6–10 °C (Fig. 4b).

Correlations of TRW chronologies with duration of the season are generally smaller than with the dates of crossing T_thr_ but have the same patterns as correlations with dates of crossing T_thr_ in spring (Fig. 4c). The sums of active temperatures in degree-days also negatively correlate with TRW of both species, with the strongest correlations observed for T_thr_ > 12 °C in the Minusinsky Bor (Fig. 4d). In the mountains, this relationship is also stronger for higher T_thr_ and has a latitudinal gradient—weakening toward the south.

## Discussion

The high heat capacity of water makes large water bodies powerful heat reservoirs that can delay and decrease in magnitude the air temperature fluctuations^9,18^. Evaporation from a vast water surface can contribute to the moisture content of the air, and breeze winds can affect the circulation of air masses and generation of precipitation^34^. Our results show significant temporal changes in the air-temperature regime in the impact zone of both the Krasnoyarsk and Sayano-Shushensky reservoirs.

A comparison of T series from stations within the KhMD showed that this zone of influence extends from the Yenisei River to the west and east within the bottom of the valley for 30–50 km. Due to the prevalence of westerly and south-westerly winds, this impact can be expected to extend somewhat farther east than west. The southern part of the KhMD and the adjacent Western Sayan foothills are influenced mainly by changes in temperature of the river water downstream of the Sayano-Shushenskoe dam. This influence is due to the discharge of deep waters, the temperature of which for deep reservoirs is maintained at about 4 °C throughout the year due to stratification^12,14^. Water temperature downstream as a result is cooler in summer and much warmer in winter than if the dam were not present. In winter this warming leads to the delay in freezing-over of the river until mid-January for up to 40 km from the dam^17,19,35^. The altered water temperatures not only lead to warmer winter air temperatures and reduced seasonal amplitude of temperature fluctuations, but also cause shifts in the onset of thermal periods critical for the growth and development of plants during the warm season. The proximity of station CHER to the riverbank downstream from the dam explains why the observed air temperature shifts there were especially large.

A less pronounced shift in temperature was observed in the central part of the valley, near the upper reaches of the Krasnoyarsk Reservoir. Here, only the surface waters of the reservoir interact with the atmosphere; air temperature follows the normal seasonal dynamics, but with fluctuations delayed and reduced in magnitude. Consistent with the lesser impact of the reservoir, the local increase in winter temperatures at MIN is half the increase at CHER. MIN is moreover located 12 km from the shoreline, albeit downwind of the reservoir. The impact of the reservoir registered at MIN is therefore damped by distance. Researchers in the 1980s noted that at MIN "the change in air temperature does not exceed the order of random discrepancies”^36^. The significant temperature shifts we observed at MIN in this study can be explained by our use of a longer period of analysis after the creation of the reservoir.

Regional and local changes in air temperature in the KhMD in the twentieth and twenty-first centuries must be interpreted in the context of natural climate change acting on a larger spatial scale. Due to the extremely continental location, winter warming at more than twice the global rate has been the imprint of recent climate change in this part of Siberia^3,4,7^. In general, the influence of reservoirs has enhanced regional warming in winter to 4–5 times the global warming rate, but hampered it in summer. As a result, spring season became extended due to the earlier transition of warming temperatures through T_thr_ = 0–8 °C. On the contrary, duration and heat supply as measured by degree-days for season with temperatures above T_thr_ = 10 °C did not change significantly.

Recent warming in the temperate latitudes of continental Asia has often been accompanied by decreased precipitation, causing an increase in the frequency and severity of droughts, depression of tree growth and even mass mortality of trees^37–39^. However, this phenomenon has not yet been observed in the KhMD and some other regions with either dendrochronological or climatic data. On the contrary, severe negative extremes in HTC and precipitation in the KhMD have recently become less frequent. One of the possible factors preventing an increase in the moisture deficit during recent warming is the mitigation of climate continentality, i.e. decrease in magnitude of temperature fluctuations^40^. This decrease at the seasonal scale reduces the rate of warming in the summer months, and at the daily scale leads to lower maximum temperatures, thus reducing potential evapotranspiration and loss of soil moisture^41,42^. Artificial water sources, such as reservoirs and irrigation systems, play a significant role in mitigation of climatic extremes, decreasing drought frequency^43^. In recent decades, such climate mitigation has been observed in the basins of many large rivers in Siberia in connection with the "boom" of hydropower: the construction of hydroelectric power plants and the creation of cascades of reservoirs^35,44^. The dynamics of extreme precipitation events have shifted toward conditions more favorable to tree growth not only close to the Yenisei River, as represented by series REG, and even at the station TASH, which presumably is the least influenced by the reservoirs. This pattern hints that parts of the KhMD far from reservoirs are not fully free from climate mitigation, but are just impacted to a lesser degree.

Conifers in the KhMD, especially from more moisture-limited sites, have a strong commonality in the dynamics of radial growth. The inter-correlation of site chronologies depends on the spatial distribution of the sites, the local conditions (e.g., soil, aspect, drainage), and the species-specific physiology and phenology of pine and larch. The contribution of geographic distance to the differences between chronologies is explained by both the high spatial heterogeneity of the precipitation field and the altitudinal-latitudinal gradients of temperature and precipitation, which provide shifts in the seasonality of the climatic response. The intensity of the climatic response is also modulated by differences in forest settings: isolated forest in the steppe zone, versus forest-steppe and subtaiga zones. Correlations of TRW chronologies are high within sites even between species due to the overlap of their similar response to exactly the same climatic fluctuations. Fundamentally similar climatic reactions of pine and larch in Siberia have been observed previously under general growing conditions^45^. On the other hand, these species have different foliage habits (evergreen/deciduous) and strategies for coping with drought: transpiration is regulated through stomata closure in pine, but proceeds actively while parts of the vascular system die back in larch^46–50^. Therefore, with an increase in the distance between sites, inter-species correlations decrease much faster than intra-species correlations between chronologies.

At sites BID and BGD in the Kuznetsk Alatau foothills we have both pine and larch chronologies, but only the pine there, like in Minusinsky Bor, respond positively to previous November precipitation. We attribute this effect to snowfall, which is low in the center of the valley (Minusinsky Bor), with a large proportion of it falling in November. In spring, photosynthesis in pine needles developed in past years is activated when the soil thaws and even earlier in hours or days with a positive air temperature^51–53^. Similar behavior was observed for other evergreen conifers^54,55^. It is likely that due to earlier activation of photosynthesis (e.g., 2–4 weeks of difference^50^), pine but not larch in our study area can use the snow as a source of moisture. The climate response of larch at BID and BGD is more similar to that of other chronologies on the Kuznetsk Alatau than to the response of pine at the center of KhMD. Strengthening and an earlier onset of the signal for climatic conditions at the end of the previous vegetative season for larch is driven by its complete annual renewal of the photosynthetic apparatus (needles) and therefore stronger dependence on the accumulation of assimilates at this time^45,48^. The Western Sayan foothills have more abundant snowfall than the other regions and a heterogeneous landscape that allows longer preservation of snow in shaded areas. These conditions apparently allow both species to use the precipitation of the second half of winter as a source of moisture. The weak pine climatic response at SHB is probably due to the lesser degree of moisture deficit in the sub-taiga zone, but the SHB chronology does have a growth pattern similar to that of chronologies at nearby sites.

Warming and an increase in the duration of the growing season, even with a continental climate, can have a positive influence on the growth of pine and larch in cold regions, such as in Yakutia^45^. In contrast, under the hotter and drier conditions of the growing season in the KhMD we find a negative influence of warming on tree growth at all sites except SHB. In other words, in the KhMD, the negative effect of increased water stress on growth is stronger than the positive effect of a longer duration of tree-ring growth and a potentially higher rate of photosynthesis and growth processes. The maximum growth responses to fluctuations in the transition dates in spring in both species are in a temperature range consistent with a meta-analysis by Rossi et al.^56^ of the crucial temperatures for the onset of xylogenesis. This indicates that the seasonality of the TRW climatic response is an indirect indicator of the phenology of cambial activity.

The timing of the autumn cooling is less important than the timing of spring warming for the growth of conifers in the KhMD. This is so despite the dependence of growth on the temperature and precipitation of the previous August–September. The lack of sensitivity to timing of temperature crossing T_thr_ in autumn suggests that the completion of radial growth of pine and larch (i.e., the cessation of cambial activity and extension of xylem cells) in KhMD is regulated more by photoperiod than by temperature. Such regulation of the completion of conifer secondary growth processes is probably characteristic of habitats with a long growing season. Similar patterns were found in the study area in the lower part of the range for Siberian spruce^57^. The negative reaction of the growth to the sum of active temperatures is maximum in the center of the valley, where winds exceeding 25 m/s are often observed in summer on flat terrain (personal observations), and are likely to exacerbate the drying effect of high temperature.

Comparison of sub-periods showed that the intensity and seasonality of the TRW climatic response in the study area changed over time. First, the TRW response to precipitation has shifted to earlier dates. This may be due to the earlier onset of photosynthesis, secondary growth, and the achievement of maximum growth rate. In contrast, the TRW response of pine to temperature (negative) has shifted to later dates. Taking into account that the delay is observed in the second half of April, when the temperatures at the sampling sites are not higher than + 5 °C, as well as the absence of this phenomenon in larch, it can be assumed that the observed effect is rather related to the early onset of primary growth and activation of pine photosynthesis. At this time, immediately after the establishment of positive temperatures, pine can use the current and previous precipitation for photosynthesis, while the drying effect of heat in this temperature range is weakly expressed (compare the positive reaction of conifers to both temperatures and precipitation in cold conditions^55^). The relationship of the shift to advanced phenology in spring is also supported by the abrupt nature of this shift and its greater severity in the areas near the reservoirs, where changes in the dates of temperature transition in the range of T_thr_ = 0–5 °C were also abrupt. It is known that bud burst and activation of photosynthesis in trees can depend not only on a certain threshold of air temperature, but also on the sum of active temperatures, photoperiod, soil temperature, the duration of the chilling requirements and the timing of snow melting^58–61^. A better understanding of the phenological triggers for larch and pine could be advanced by more physiological research using the study area as a testing ground for its uniquely high rate of climatic changes and diverse forest settings.

The weak connection of pointer years in the growth of conifers with the seasonal climatic factors examined is not surprising, as extreme deviations in growth are often caused by short-term climatic extremes (dry periods, heavy rainfall, heat waves, frosts, etc.^62,63^) that might not force a significant deviation in climate variables summarized for the complete growing season. Regional-scale fires and pest outbreaks can also cause pointer years in TRW, although these factors are not independent of climate: droughts make fires more frequent and increase the rate of fire spreading; and warm dry weather is favorable for egg quantity and survival for many insect pest species. Therefore, the most likely factor acting over large distances and multiple species is climate. Carryover biological processes can blur the relationship of pointer years to climate, as the formation of the annual ring depends not just on climate of the current growth season, but to some degree on climatic conditions in the second half of the previous growing season, especially for larch. Despite the complicating factors, our comparison of the frequency of favorable/unfavorable seasonal climate and positive/negative pointer years does suggest that the smoothing of climatic fluctuations after the creation of reservoirs coincides with a decrease in the frequency of growth depressions and an increase in the frequency of wide rings. A curious phenomenon is the apparent absence of severe growth depressions during the 1998–2008 period of very hot vegetative seasons. A possible explanation is the abundant precipitation during that period, or at least low frequency of very dry years. This attests to the importance of precipitation for the growth dynamics of the two tree species.

## Methods

### Instrumental climatic data analysis

For monthly resolution of climate dynamics we used time series of monthly average air temperature (T), and total monthly precipitation (P), at state climatic stations near the center and toward the fringes of the valley (Table 1). Daily P and T data were available for stations TASH, MIN, and CHER (Fig. 1). Data coverage of station time series is shown in Supplementary Fig. S6. Wind conditions in the KhMD were summarized with a wind rose of monthly and annual wind directions’ repeatability data from MIN station, near the center of the valley. Climatic data were obtained from All-Russian Research Institute of Hydrometeorological Information, World Data Centre (RIHMI-WDC; http://meteo.ru/data).

Monthly climatic series from near boundaries of the KhMD were averaged into a regional series, REG, to represent climate dynamics with minimal impact of reservoirs (TASH data were used for this purpose at daily resolution). To detect and quantify climatic effects of dams, it was necessary to identify subsets of climate stations subject to and free from such effects. The station whose climate was least likely to be distorted by environmental changes due to development of the reservoirs is TASH, which is far removed from (100 km and more) and upwind of both reservoirs (see wind rose in Fig. 1). To identify other stations with a minimally distorted regional climate signal, we computed the difference series of the monthly climate variables (*d*P and *d*T) between each station and TASH. If *d* is temporally stable, the station record is assumed to represent larger-scale regional climate variations and to be relatively unaffected by the dams and other local natural and anthropogenic landscape change^64,65^. Stability was tested by a *t*-test^66^ of the difference of sub-period means of *d* before and after construction of the dams. The two sub-periods for the test were 1936–1967 and 1980–2014: the first is before formation of the Krasnoyarsk Reservoir and the second is after formation of the Sayano-Shushensky Reservoir. The transitional period 1968–1979 was excluded from consideration in order to simplify and homogenize the analysis. Results were used to identify stations and seasons with significant local climatic shifts relative to station TASH (Supplementary Table S4). Results of *t*-test for P were chaotic, with no consistent interpretable pattern. BEYA station, however, was excluded because of absence of P series before 1967. Results for T suggest a clear stratification of stations: the difference in means of *d*T in the two sub-periods is significant for three or more months at stations BGD, MIN, KUR, ERM, and CHER. The shift in *d*T for these stations is consistent with reservoir influence: mainly warming in April-August and cooling in September–March. In contrast, mostly stable climate dynamics (non-significant *t*-test) were found for stations SHIRA, UYB, IDR and KAR. Some scattered missing data (Supplementary Fig. S6) were estimated for these stations by linear regression of the respective P or T series on corresponding data at TASH for the period of overlapping data. After that, these series were combined with TASH using a robust mean to build a regional average climate series, REG, with minimized reservoir impact. All five stations for the regional average are located at a considerable distance from the Yenisei River, closer than the other stations to the western and eastern boundaries of the Khakass-Minusinsk Depression (Fig. 1). Since all stations used to estimate regional climate, except TASH, have only monthly data available, TASH series were used in analysis of climate dynamics with minimal impact of reservoirs at daily resolution instead of REG.

Stations MIN and CHER, which are nearest the reservoirs, were used to represent climate variations modified by the reservoirs at both monthly and daily resolutions. Time plots of the monthly spatial differences series MIN-REG and CHER-REG (*d*P and *d*T) were used to clarify the onset of the reservoir influence on climate. Occurrence of extremes was explored in seasonal P and T averaged for May–September. Extremes were defined as values exceeding 1.28 and 1.645 standard deviations from the mean, corresponding to 10% and 5% probability points of the normal distribution^66^. Means and standard deviations were computed over the base 30-year period 1951–1980 (the earliest climatic normal^67^ available for all considered series).

Daily T series were used to calculate derivative characteristics: dates of stable temperature crossing of T_thr_ = 0 to 12 °C with 0.5 °C increment in autumn and spring, duration of season between these dates, and the cumulative sum of temperatures above T_thr_ = 0 to 21 °C during the season. According to agroclimatic convention in Russia^68^, the date of stable temperature crossing of threshold T_thr_ in spring is the first day of the first continuous period of daily T > T_thr_ with Σ|T–T_thr_| for this period being more than for any subsequent period of T < T_thr_; in autumn, it is calculated similarly but with reversed sign (i.e., T < T_thr_). Time series of these dates were calculated automatically from daily T series at TASH, MIN, and CHER stations in program created by Bureeva M.A. (program unregistered). Duration of season with temperatures above T_thr_ = 0–12 °C was calculated as difference between spring and autumn dates of temperature stably crossing these thresholds. Sum of temperatures above T_thr_ (degree-days) was calculated for these seasons as Σ(T–T_thr_) for days with T > T_thr_. To estimate actual temperature values at the sampling sites, values of T_thr_ were shifted by the corrections calculated as elevational differences between stations and sampling sites, multiplied by the average elevational temperature lapse rate in the atmosphere: 0.65 °C per 100 m^69,70^. Crossing dates, duration of season between them, and temperature sums above T_thr_ were compared for sub-periods before and after possible reservoir influence.

The hydrothermal regime of the basin during the warm period (May to September) was summarized for REG and stations TASH, MIN and CHER by the Selyaninov hydrothermal coefficient (HTC = 10·ΣP/ΣT for days with T > 10 °C^71^). The HTC is an agroclimatic index of moisture availability to plants, and has previously been applied to study climatic factors related to tree-ring variation in the study region^72^. To compute the HTC for REG, the sum of average daily temperatures was estimated as the mean monthly T multiplied by number of days in the month. We used conventional empirical thresholds for extremes of seasonal aridity: HTC < 1.0 (arid), 1.0 ≤ HTC < 1.25 (semiarid), 1.75 < HTC ≤ 2.0 (subhumid), and HTC > 2.0 (humid).

### Development and analysis of TRW chronologies

Wood samples (cores) of Scots pine and Siberian larch were collected in 2012–2019 from habitats experiencing moisture deficit (Fig. 1, Table 1): isolated forest Minusinsky Bor within the steppes of the central part of the KhMD (sampling sites ZSH, MMIN, TAR, MNIC), forest-steppe near the borders of the valley in the foothills of the Kuznetsk Alatau (BGD, BID, KAM, KAZ) and the Western Sayan Mountains (KALY, MAY), and sub-taiga forest higher up the slope of the Western Sayan (SHB). In mountain habitats we preferred sites on south-facing slopes, where the moisture deficit is accentuated. Field work was performed in compliance with relevant regulations on collection of plant material. Tree species involved in the study are not endangered or protected, and collection of their samples is not restricted or forbidden. Site SHB is located in the protected natural area (National Park “Shushensky Bor”), sampling there was carried out with permission from park administration. Field work and sample processing were carried out using standard dendrochronological methods^73^, with device LINTAB 5 and program TSAPwin^74^ for measurement, program COFECHA^75,76^ for cross dating, and program ARSTAN^77^ for standardizing and averaging. Trends associated with age (represented by negative exponential or linear functions) and an autocorrelation component were identified and removed in standardizing measured widths to dimensionless indices. Elimination of autocorrelation was performed to highlight climatic signal, since T and P in the study area do not have significant inter-annual autocorrelation. Local residual TRW chronologies for each species and sampling site were obtained by averaging standardized indices over trees with a bi-weight mean^73^.

Local chronologies were characterized by descriptive statistics (e.g., mean and standard deviation), and by the mean sensitivity (*sens*), defined as the average over the entire chronology of the ratio of the difference of successive TRW indices to their mean^78^. The strength of common variation within the set of series comprising a chronology was summarized by the average between-series correlation (*r-bar*^73^). The strength of common growth signal was also judged by the expressed population signal (EPS^79^). A threshold of EPS > 0.85 was used to delineate parts of chronologies with sample number sufficient to represent the population growth signal. For climatic analysis, chronologies were truncated to eliminate any leading years with EPS < 0.85.

The climate signal in TRW was summarized by pairwise Pearson correlations of chronologies with various climate variables: monthly P and T; daily P and T in a 21-day moving window, and derivative temperature characteristics. The correlations were calculated both for the entire overlapping periods between each particular instrumental climatic series and chronology (see Supplementary Fig. S6, Supplementary Table S1 for cover periods), and for shorter sub-periods. The “pointer years” (extremes of TRW) were defined in the same way as the climate extremes, except that means and standard deviations for normal probability points were computed over the full length of chronologies.

## Conclusion

Our findings confirm the impressive regional scale of climate modification associated with building of large dams on Siberian rivers, and highlight the consequent impacts on forest ecosystems. Results also underscore the complexity of delineating anthropogenic impacts against the background of rapidly occurring natural climate change and, as in other studies^45,50,80^, emphasize that impacts on tree-growth in particular can be highly species-specific. Effects of climate modification on tree growth in the KhMD appears to be species-specific, with reservoir influence on growth extending further from the reservoir for pine than for larch. Reservoir influence may therefore modulate changes in species composition of forests with continued warming. The unexpected lack of tree-growth suppression in the recent decade or so of very warm vegetative seasons is probably explained by the offsetting of extreme heat by adequate precipitation over that period. The reservoirs may also have had some buffering effect on drought stress in trees by providing additional atmospheric moisture and cooling on the hottest days. If dry conditions were to return, however, it is likely that tree growth will eventually suffer from regional warming.

## Supplementary Information

Supplementary Information.

## Data Availability

Climatic data were obtained from RIHMI-WDC (http://meteo.ru/data). Dendrochronological data are available from the corresponding author upon reasonable request.
