# Supplementary material for: Climate change and tree growth in the Khakass-Minusinsk Depression (South Siberia) impacted by large water reservoirs
Source: Sci Rep. 2021 Jul 12;11:14266. doi: 10.1038/s41598-021-93745-0 (PMC8275609; doi:10.1038/s41598-021-93745-0)
Supplement: Supplementary file 1 — Supplementary Information. [file 41598_2021_93745_MOESM1_ESM.pdf]

Climate change and tree growth in the Khakass-Minusinsk Depression (South Siberia) impacted by large water reservoirs

Zhirnova D.F., Belokopytova L.V., Meko D.M., Babushkina E.A., Vaganov E.A.

Supplementary materials

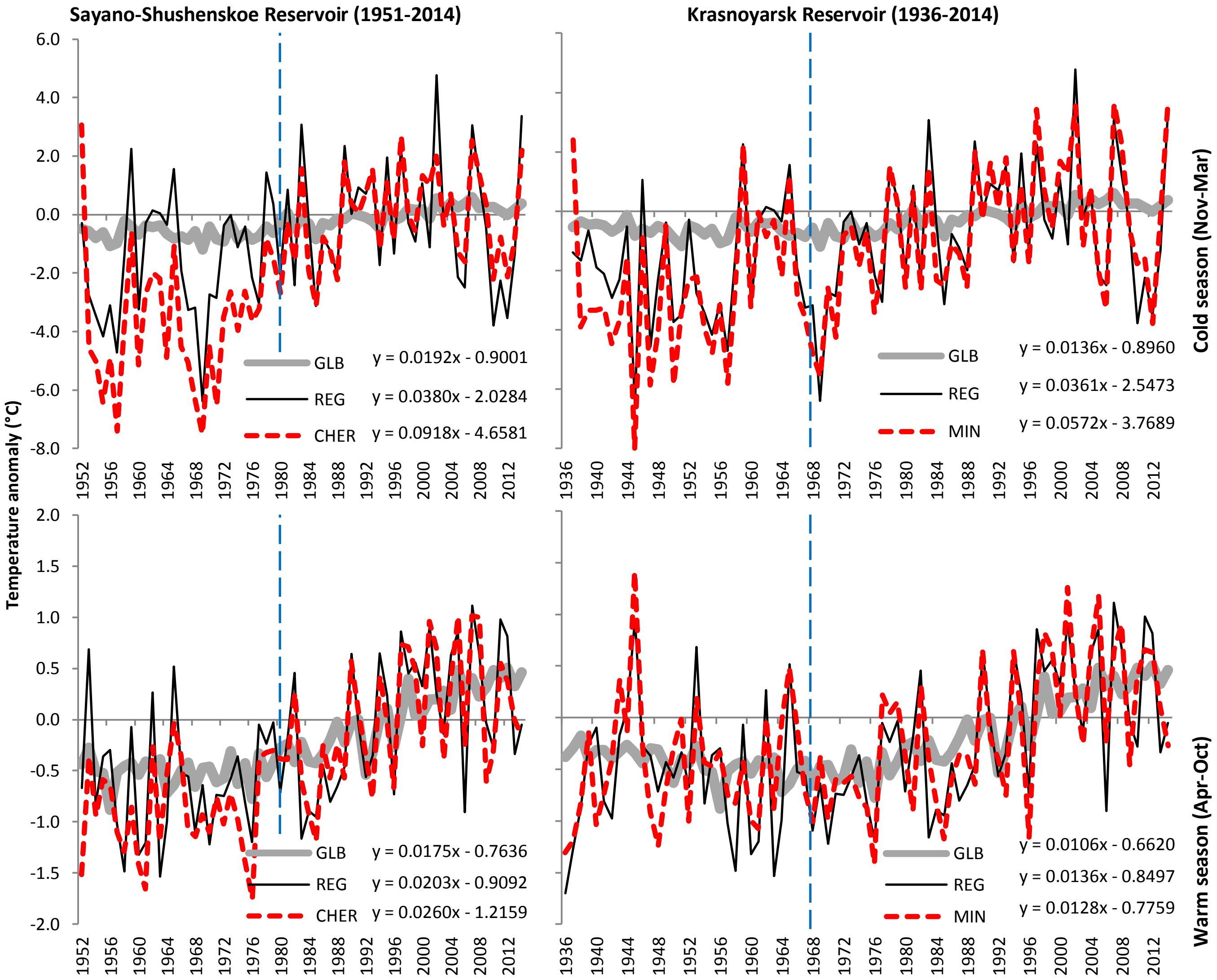

Fig. S1. Climatic dynamics at different scales: long-term trends of temperature anomalies (absolute values minus mean of base period 1981-2010) for warm (top panels) and cold (bottom panels) seasons: Northern Hemisphere average temperature from CRU TS (GLB; https://crudata.uea.ac.uk/cru/data/hrg/cru_ts_4.04/), regional temperature averaged from several stations (REG), and local temperature at stations near reservoirs (CHER, MIN). Equations show linear trends, where the first coefficient is warming rate in °C per year. Vertical dashed lines represent year of the first turbines launching in the respective dam

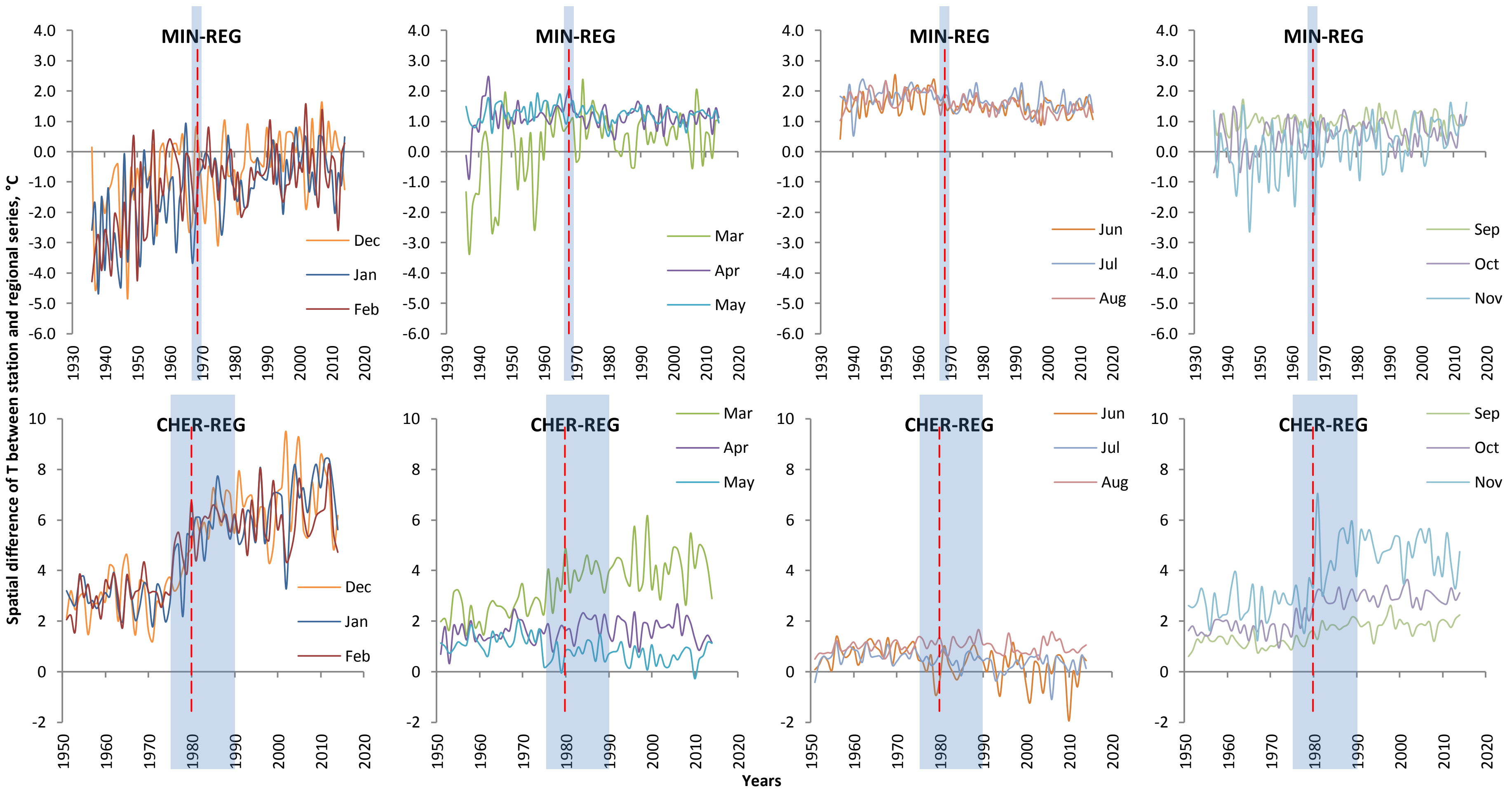

Fig. S2. Shifts of local monthly temperatures near Krasnoyarsk Reservoir (MIN – Minusinsk station) and Sayano-Shushenskoe Reservoir (CHER - Cheryomushki station) from regional pattern REG. Marked are periods of filling reservoirs (1967-1970 for Krasnoyarsk Reservoir and 1975-1990 for Sayano-Shushenskoe Reservoir) and the first turbines launching (beginning of 1969 and 1980, respectively)

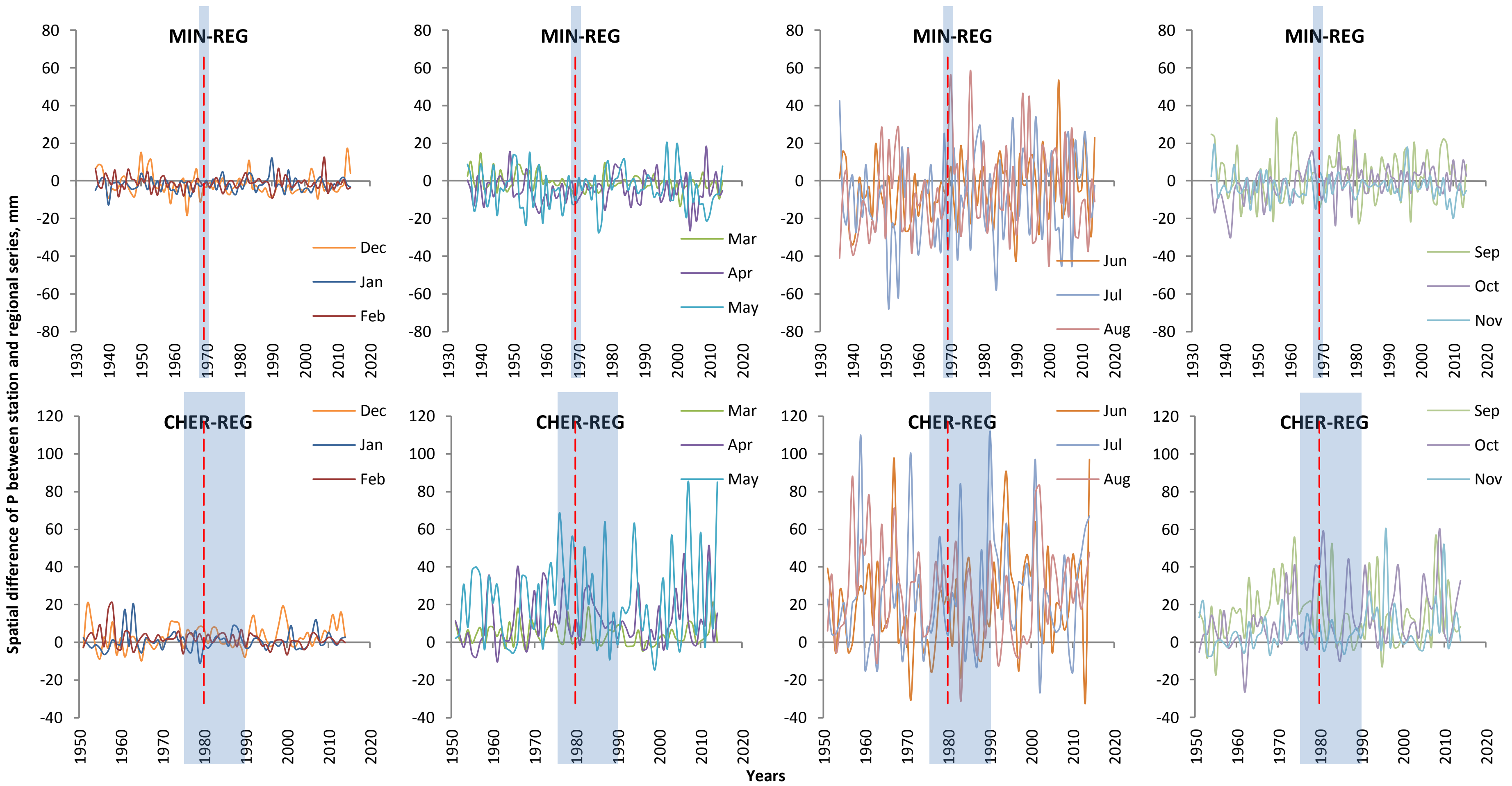

Fig. S2. Continued. Shifts of local monthly precipitation near Krasnoyarsk Reservoir (MIN – Minusinsk station) and Sayano-Shushenskoe Reservoir (CHER – Cheryomushki station) from regional pattern REG. Marked are periods of filling reservoirs (1967-1970 for Krasnoyarsk Reservoir and 1975-1990 for Sayano-Shushenskoe Reservoir) and the first turbines launching (beginning of 1969 and 1980, respectively)

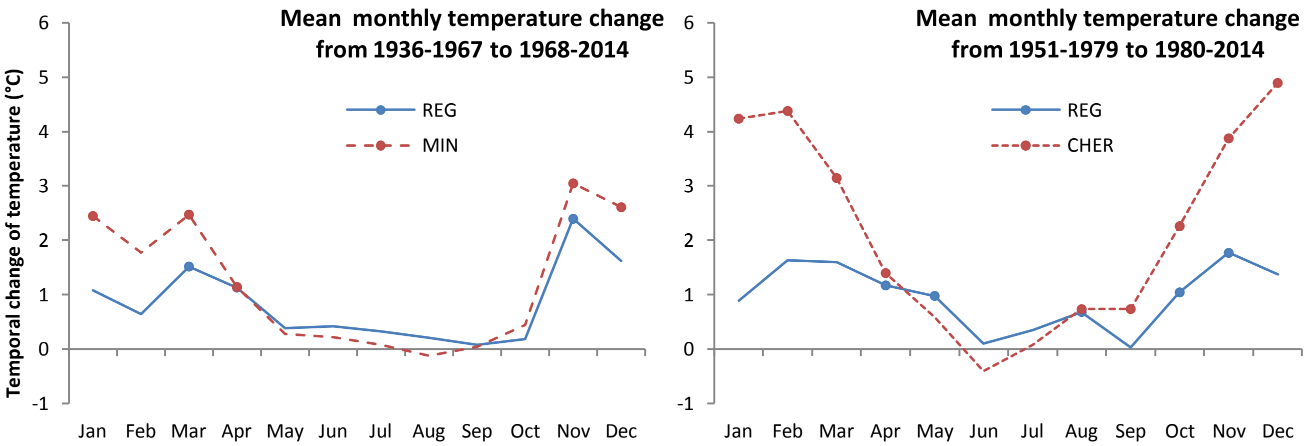

Fig. S3. Differences between sub-period mean values of monthly temperatures regionally (REG) and near reservoirs (MIN, CHER) before and after launching of the turbines in the respective dams. Dots mark significance at $p<0.05$

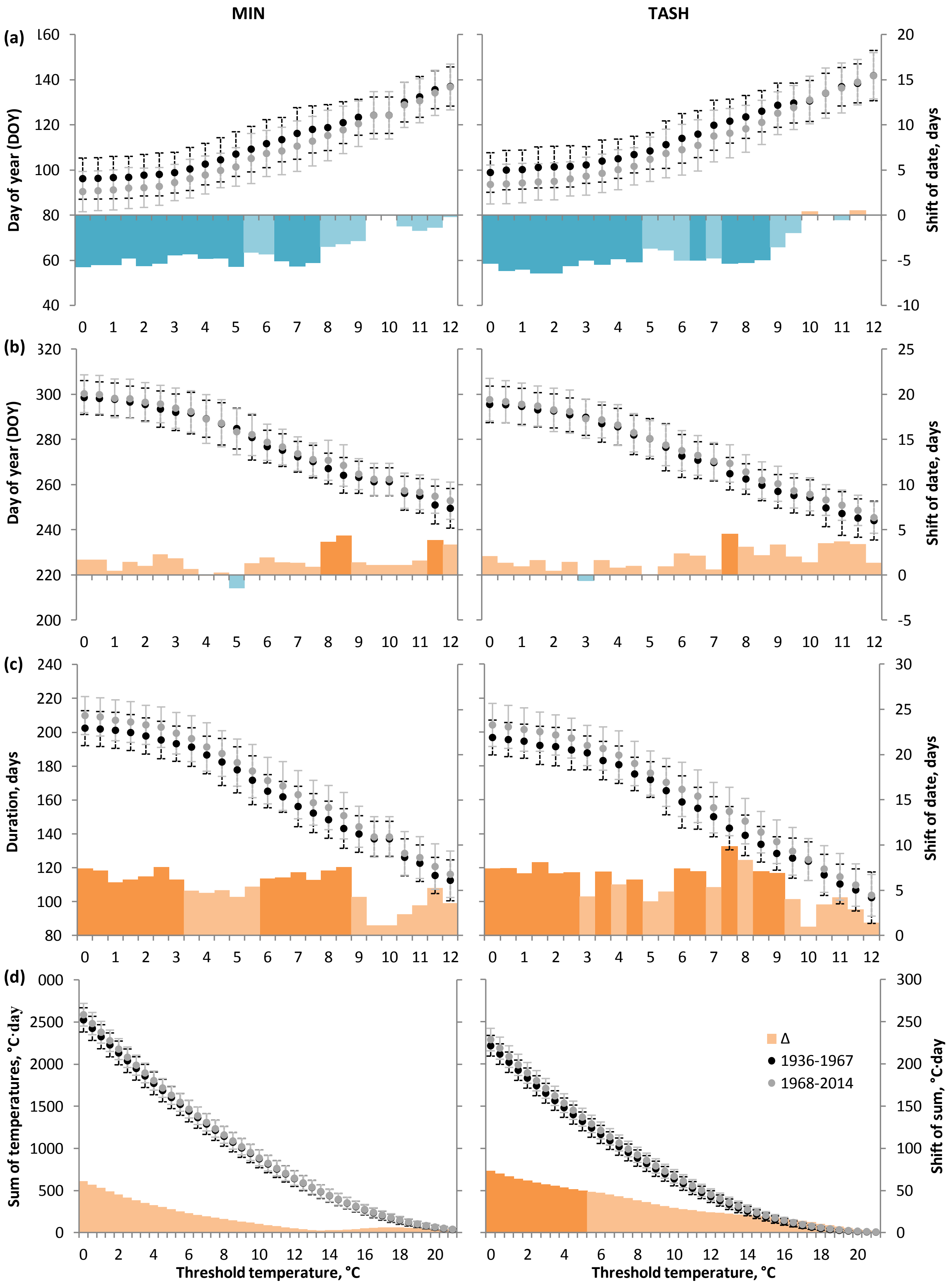

Fig. S4. Comparison between sub-periods before and after 1968 for temperature-related climatic variables at MIN and TASH stations: **(a)** dates of temperature crossing threshold values in spring; **(b)** the same in autumn; **(c)** duration of interval with temperatures above threshold; **(d)** sum of temperatures above threshold. Dots are mean values and whiskers are standard deviations for sub-period, bars are temporal differences between mean values for two sub-periods (dark shades mark significant shifts at $p<0.05$)

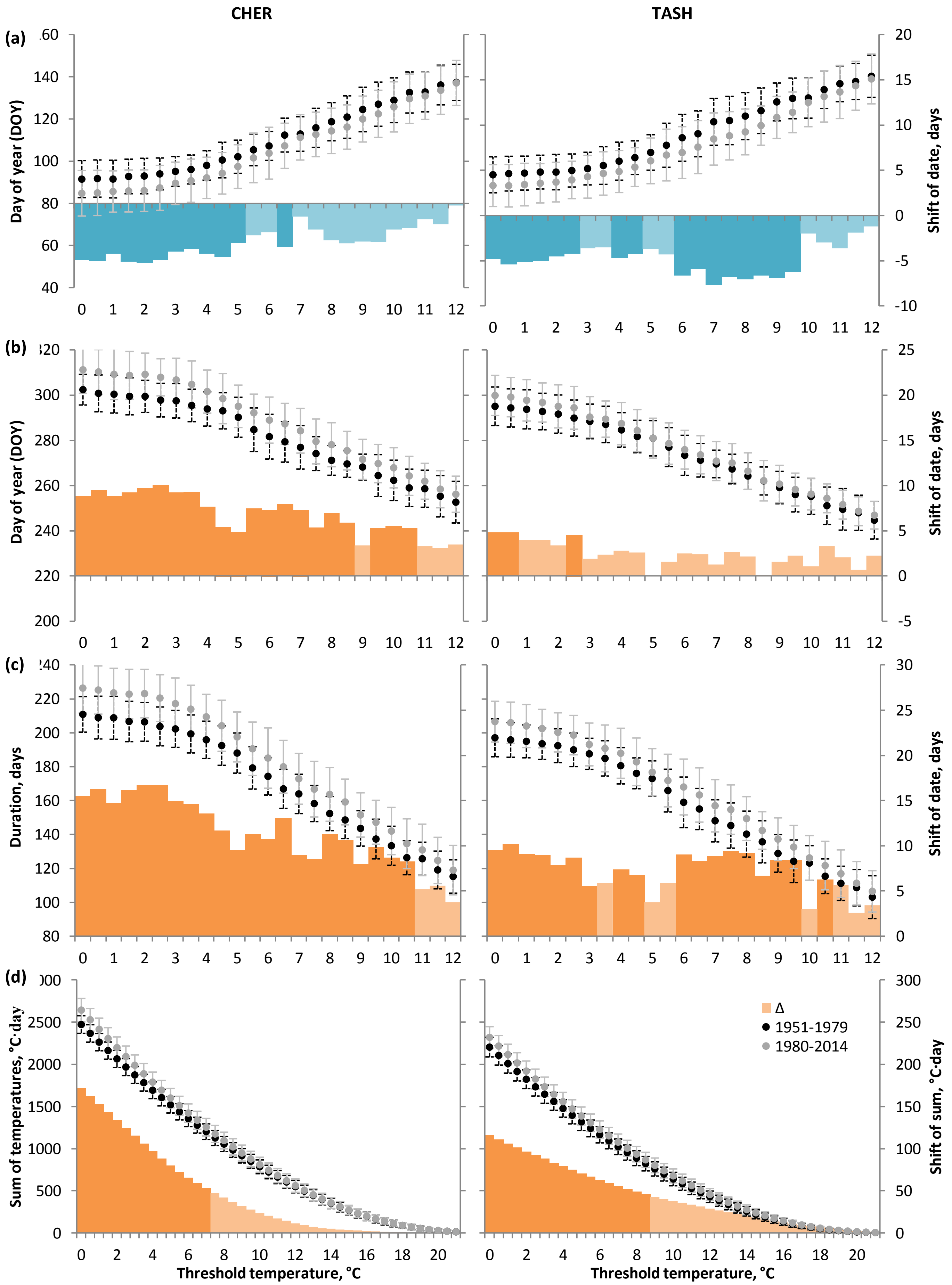

Fig. S4. Continued. The same for sub-periods before and after 1980 at CHER and TASH stations

Table S1. Statistics of residual TRW chronologies. The last four columns are the standard deviation (*stdev*), mean sensitivity (*sens*), mean between-series correlation (*r-bar*), and beginning year of period with expressed population signal (*EPS*) above 0.85 (Wigley et al., 1984)

Site & species	Sample				Chronology			
	No. of cores	Cover period (years)	Length (years)	mean TRW (mm)	*stdev*	*sens*	*r-bar*	*EPS*>0.85 from year
Pinus sylvestris (PS)								
ZSH_PS	144	1881-2017	137	2.14	0.25	0.30	0.31	1916
MMIN_PS	40	1847-2013	167	2.32	0.19	0.22	0.41	1898
TAR_PS	34	1914-2013	100	2.02	0.19	0.23	0.49	1926
MNIC_PS	29	1872-2013	142	2.29	0.20	0.24	0.43	1922
BGD_PS	80	1847-2018	172	1.28	0.26	0.31	0.42	1876
BID_PS	16	1874-2018	145	1.97	0.28	0.34	0.56	1911
KAZ_PS	67	1767-2013	247	1.32	0.31	0.39	0.53	1798
KALY_PS	13	1947-2012	66	2.85	0.20	0.22	0.33	1951
MAY_PS	27	1947-2017	71	2.55	0.23	0.29	0.46	1948
SHB_PS	31	1914-2015	102	2.69	0.11	0.13	0.30	1925
Larix sibirica (LS)								
BGD_LS	44	1845-2019	175	0.94	0.31	0.37	0.47	1882
BID_LS	68	1704-2019	316	1.23	0.29	0.34	0.45	1815
KAM_LS	61	1710-2019	310	0.92	0.35	0.39	0.44	1759
KAZ_LS	20	1835-2013	179	2.09	0.46	0.52	0.47	1889
KALY_LS	13	1946-2012	67	2.50	0.30	0.38	0.51	1951

Table S2. Correlations between TRW chronologies (correlation period varies according to the overlap of each pair of chronologies within 1936-2014). Colored correlations (gradient according to values) are significant ($p<0.05$)

	BGD_PS	BID_PS	ZSH_PS	MMIN_PS	TAR_PS	MNIC_PS	KAZ_PS	KALY_PS	MAY_PS	SHB_PS	BGD_LS	BID_LS	KAM_LS	KAZ_LS	KALY_LS
BGD_PS	**1.00**														
BID_PS	**0.63**	**1.00**													
ZSH_PS	**0.60**	**0.52**	**1.00**												
MMIN_PS	**0.58**	**0.55**	**0.64**	**1.00**											
TAR_PS	**0.56**	**0.51**	**0.63**	**0.95**	**1.00**										
MNIC_PS	**0.50**	**0.41**	**0.65**	**0.77**	**0.72**	**1.00**									
KAZ_PS	**0.66**	**0.50**	**0.65**	**0.57**	**0.55**	**0.51**	**1.00**								
KALY_PS	**0.43**	**0.38**	**0.32**	**0.39**	**0.43**	**0.42**	**0.32**	**1.00**							
MAY_PS	**0.45**	**0.37**	**0.48**	**0.34**	**0.37**	**0.43**	**0.55**	**0.52**	**1.00**						
SHB_PS	**0.32**	0.19	**0.26**	**0.32**	**0.32**	**0.40**	**0.39**	**0.44**	**0.43**	**1.00**					
BGD_LS	**0.73**	**0.44**	**0.44**	**0.30**	**0.30**	**0.29**	**0.58**	0.21	**0.36**	**0.28**	**1.00**				
BID_LS	**0.52**	**0.58**	**0.52**	**0.38**	**0.40**	**0.26**	**0.46**	0.19	**0.44**	0.09	**0.63**	**1.00**			
KAM_LS	**0.54**	**0.40**	**0.53**	**0.27**	**0.30**	0.20	**0.50**	0.22	**0.38**	0.12	**0.70**	**0.73**	**1.00**		
KAZ_LS	**0.41**	**0.29**	**0.43**	**0.37**	**0.39**	**0.30**	**0.73**	0.12	**0.38**	0.22	**0.50**	**0.43**	**0.59**	**1.00**	
KALY_LS	**0.42**	**0.30**	**0.45**	**0.35**	**0.39**	**0.41**	**0.54**	**0.55**	**0.74**	0.22	**0.45**	**0.49**	**0.48**	**0.52**	**1.00**

Table S3. Correlations of TRW chronologies with monthly temperature and precipitation from previous July to current August. Asteriscs (*) mark months of the previous year. Colored correlations (green, positive; red, negative) are significant ($p<0.05$)

Month	Pine TRW chronologies										Larch TRW chronologies				
	BGD_PS	BID_PS	ZSH_PS	MMIN_PS	TAR_PS	MNIC_PS	KAZ_PS	KALY_PS	MAY_PS	SHB_PS	BGD_LS	BID_LS	KAM_LS	KAZ_LS	KALY_LS
Temperature															
Jul*	0.03	0.14	0.00	0.21	0.17	**0.24**	0.01	-0.07	-0.17	0.02	-0.12	-0.21	**-0.27**	-0.16	-0.15
Aug*	**-0.28**	-0.20	**-0.28**	-0.18	**-0.23**	-0.17	**-0.22**	**-0.47**	**-0.45**	**-0.31**	**-0.29**	**-0.30**	**-0.37**	**-0.31**	**-0.36**
Sep*	**-0.30**	**-0.32**	**-0.25**	-0.22	**-0.25**	-0.07	-0.14	-0.05	-0.08	-0.17	**-0.29**	**-0.38**	**-0.34**	-0.18	0.00
Oct*	-0.18	-0.10	-0.10	-0.13	-0.16	-0.04	0.07	0.10	0.06	0.04	-0.05	-0.07	-0.05	0.16	0.06
Nov*	-0.04	-0.10	0.01	-0.08	-0.08	0.04	0.07	-0.07	-0.08	0.02	0.09	0.10	0.20	**0.27**	0.01
Dec*	**-0.25**	-0.13	-0.11	-0.16	-0.18	-0.07	-0.12	-0.04	-0.12	-0.07	-0.02	-0.11	0.08	0.01	0.02
Jan	-0.15	0.13	0.05	0.07	0.16	0.08	-0.02	0.22	0.19	0.11	-0.13	-0.01	0.00	0.06	0.07
Feb	-0.03	-0.06	0.06	0.18	0.17	**0.28**	0.04	0.04	0.14	-0.04	-0.02	-0.10	0.00	0.16	0.17
Mar	-0.07	-0.05	-0.06	-0.10	-0.12	-0.02	-0.08	0.02	0.04	0.08	0.02	-0.09	-0.04	-0.15	0.00
Apr	-0.14	**-0.23**	-0.17	-0.05	-0.04	0.07	-0.12	-0.02	0.03	**0.29**	-0.06	**-0.23**	**-0.25**	-0.07	-0.08
May	**-0.35**	-0.15	**-0.24**	-0.21	**-0.24**	-0.15	**-0.31**	**-0.30**	**-0.35**	**-0.29**	**-0.37**	**-0.24**	**-0.38**	**-0.31**	**-0.34**
Jun	-0.19	-0.20	-0.06	**-0.30**	**-0.34**	**-0.23**	**-0.30**	-0.14	-0.07	-0.04	-0.11	-0.19	**-0.23**	**-0.30**	-0.12
Jul	**-0.29**	-0.19	**-0.36**	**-0.28**	**-0.27**	**-0.28**	-0.11	-0.21	**-0.25**	-0.14	-0.13	-0.17	-0.16	-0.10	-0.20
Aug	-0.20	**-0.24**	**-0.24**	**-0.25**	**-0.28**	**-0.27**	-0.20	-0.23	-0.11	-0.10	-0.12	-0.13	0.00	-0.10	-0.10
Precipitation															
Jul*	0.18	0.10	0.15	-0.07	-0.08	0.04	0.18	0.14	**0.29**	0.08	0.19	0.18	**0.27**	0.12	**0.28**
Aug*	**0.32**	**0.35**	**0.46**	**0.42**	**0.41**	**0.36**	**0.28**	**0.36**	**0.44**	**0.23**	**0.23**	**0.31**	**0.44**	**0.26**	**0.43**
Sep*	0.22	**0.32**	**0.26**	**0.24**	**0.23**	0.19	**0.28**	0.08	0.20	0.20	0.06	**0.23**	0.10	0.21	0.13
Oct*	0.05	0.08	0.05	0.21	0.20	0.07	0.22	0.05	-0.01	-0.15	0.10	-0.01	0.06	**0.26**	0.17
Nov*	**0.34**	**0.33**	**0.48**	**0.49**	**0.44**	**0.46**	0.18	0.05	0.12	0.13	0.20	0.17	0.15	0.18	0.03
Dec*	0.15	0.20	0.08	0.17	0.11	0.09	0.16	-0.07	-0.07	0.04	0.18	0.12	0.06	0.17	-0.04
Jan	0.07	0.12	**0.22**	0.18	0.09	0.11	0.03	**0.32**	**0.32**	0.18	-0.02	0.04	-0.05	0.04	**0.31**
Feb	0.20	0.10	**0.33**	0.18	0.10	0.20	0.20	0.19	**0.31**	0.19	0.01	0.03	0.09	**0.25**	**0.25**
Mar	-0.04	-0.05	0.00	0.12	0.06	0.12	0.07	**0.27**	**0.26**	0.22	-0.16	-0.17	-0.02	0.02	**0.33**
Apr	0.15	**0.23**	**0.24**	0.03	0.03	0.01	**0.25**	0.12	0.22	0.10	0.11	0.12	0.07	0.22	0.20
May	**0.31**	**0.25**	**0.46**	**0.36**	**0.38**	**0.36**	**0.27**	**0.30**	**0.43**	**0.33**	**0.35**	**0.24**	0.19	0.06	**0.34**
Jun	**0.29**	**0.23**	**0.32**	**0.32**	**0.31**	**0.26**	**0.42**	0.00	0.09	-0.01	**0.31**	**0.34**	**0.26**	**0.28**	0.22
Jul	0.16	0.17	0.21	0.20	0.18	**0.26**	0.01	**0.29**	0.16	0.19	0.05	0.11	0.06	-0.11	0.17
Aug	-0.09	0.07	-0.07	-0.10	-0.09	-0.03	0.09	-0.08	-0.08	-0.02	-0.19	-0.21	**-0.30**	-0.06	-0.09

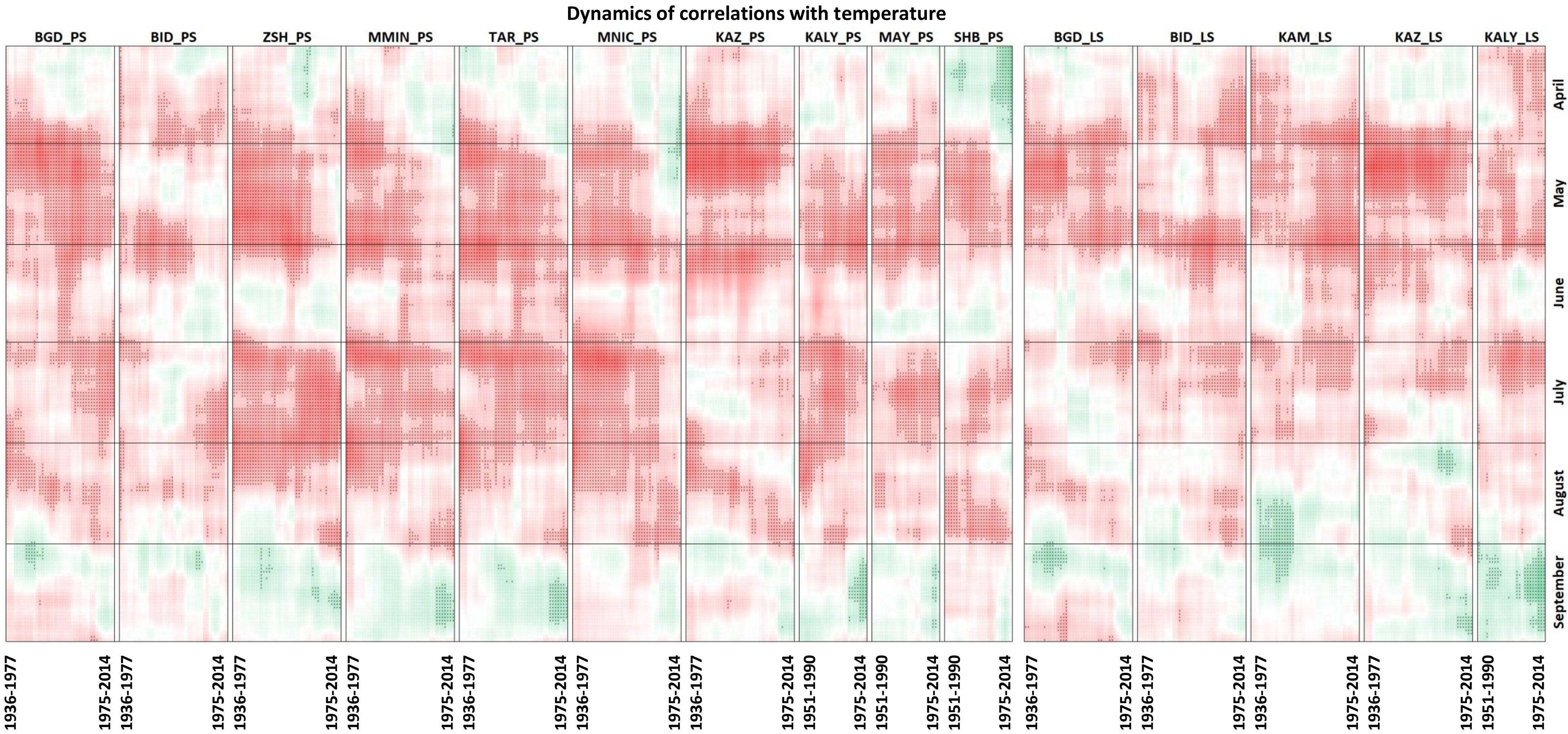

Fig. S5. Moving correlations between TRW of pine and larch from semiarid conditions and temperature (40-year window 1-year step on the horizontal axis, 21-day window 1-day step on the vertical axis). Correlation values are represented by gradient scale, where positive correlations are in green, negative ones are in red, zero is white. Significant correlations at $p<0.05$ are marked with dots

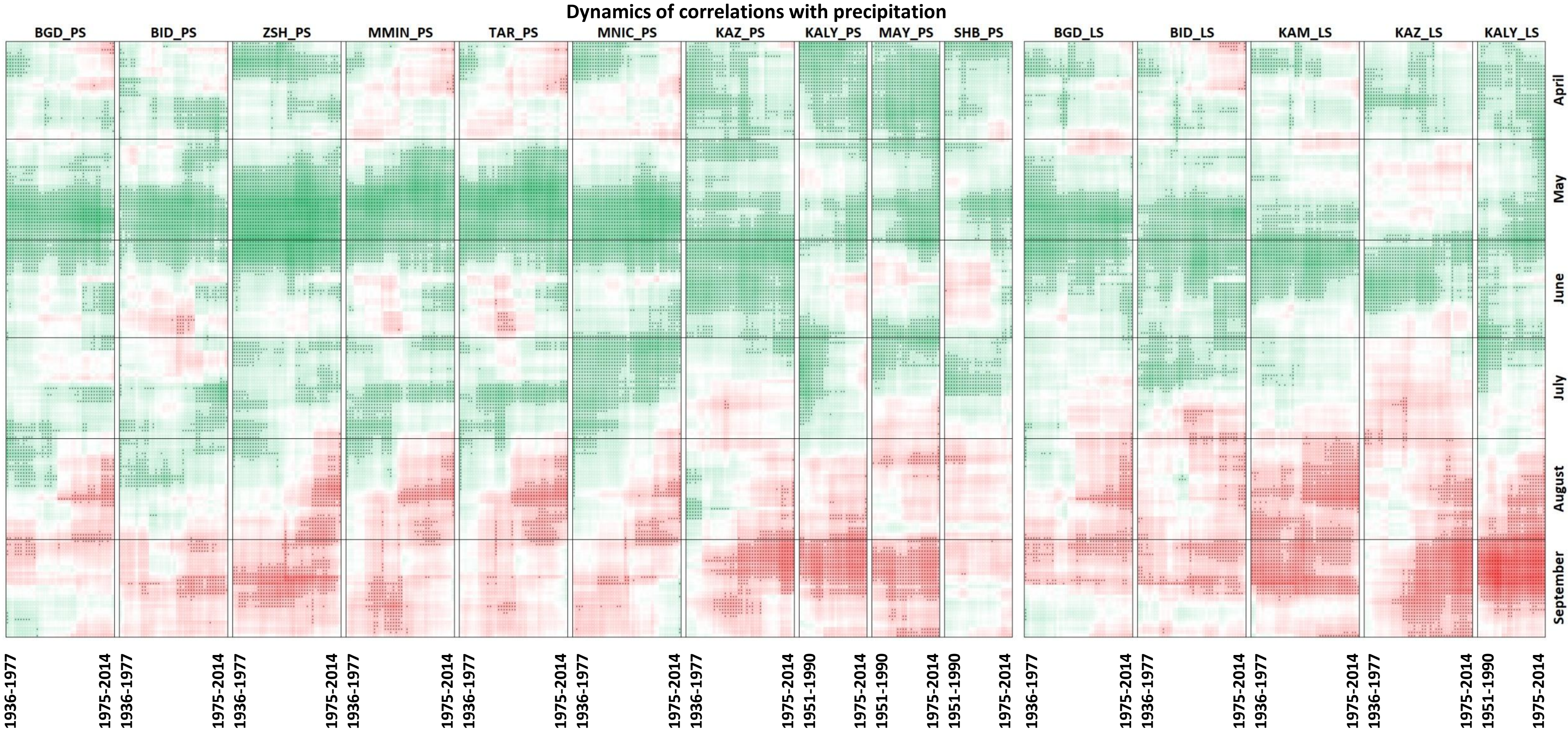

Fig. S5. Continuation: the same for precipitation

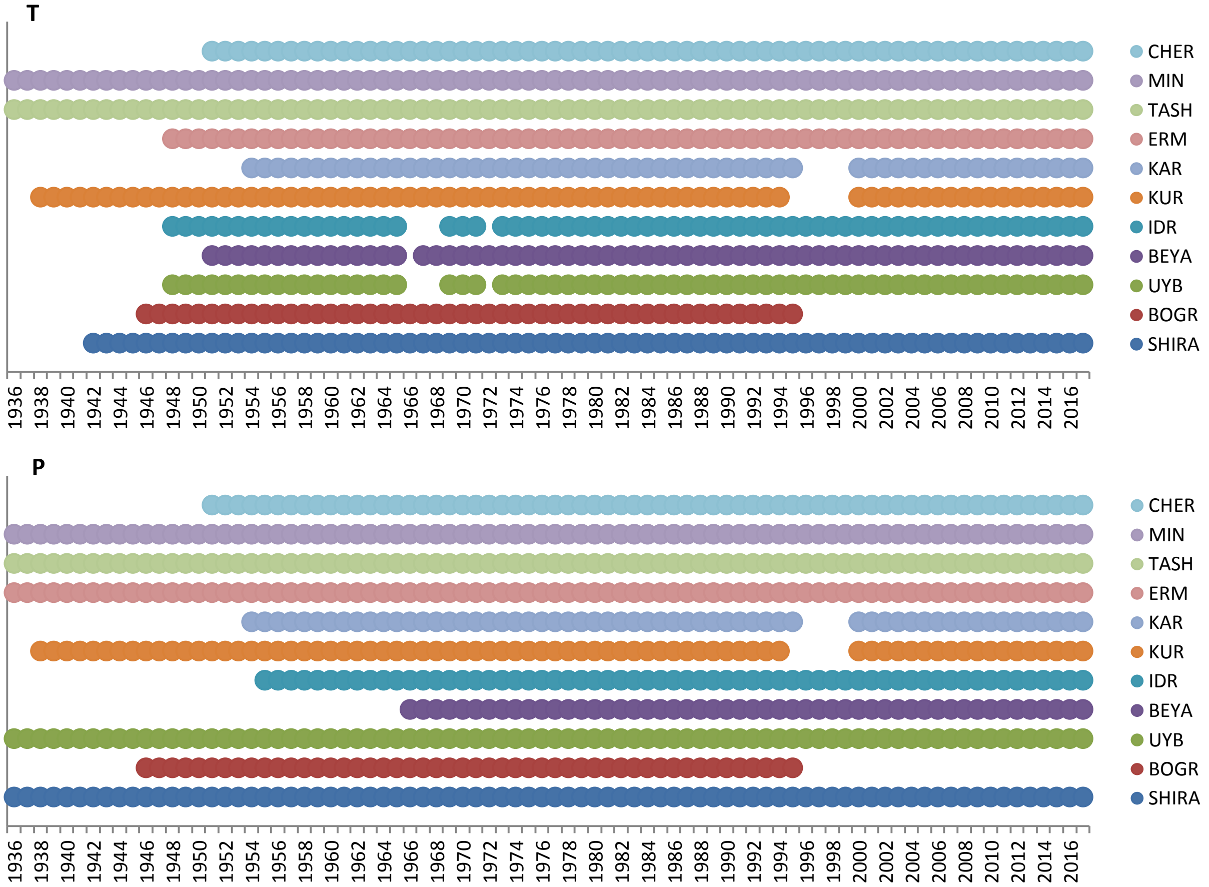

Fig. S6. Cover periods and missing data of climatic series. Station codes see Table 1

Table S4. Differences between monthly climate (P and T) at various climatic stations and TASH over 1936-1967 (numerator) and 1980-2014 (denominator) sub-periods. Colored stations have the most substantial shift of the temperature regime or are not suited for assess of climate before reservoir building. Significant shifts of differences in climatic variables are bold and shaded with red (if climate became hotter and/or drier) or blue (cooler/wetter)

Station	Jan	Feb	Mar	Apr	May	Jun	Jul	Aug	Sep	Oct	Nov	Dec
	Temperature, $dT = T_{station} - T_{TASH}$ (°C)											
SHIRA	0 / 0.5	**-0.7 / 0.3**	**-1.3 / -0.4**	-0.7 / -0.9	-0.1 / -0.4	0.3 / 0.3	0.5 / 0.4	0.2 / 0.2	0.2 / 0.3	0.1 / 0	-0.9 / -0.3	-0.5 / 0.1
BOGR	**-2.6 / -0.7**	-1.9 / -1.3	-0.2 / -1.5	**0.8 / -0.5**	**1.4 / 0.6**	**1.9 / 1.4**	2 / 2.1	1.8 / 2	**1.2 / 2**	**0.3 / 1.7**	**-1.1 / 1.8**	**-1.9 / 0.2**
UYB	**-0.6 / 0.5**	0.1 / 0.4	-0.5 / -0.2	-0.6 / -0.6	-0.3 / -0.3	0 / 0.2	**0 / 0.2**	-0.1 / 0	-0.3 / -0.4	-0.9 / -0.9	-0.9 / -1	-0.5 / -0.5
BEYA	2 / 2.3	1.7 / 2	**0.7 / 1.1**	0.5 / 0.5	0.8 / 0.6	0.6 / 0.7	0.7 / 0.7	0.8 / 0.7	0.7 / 0.7	0.8 / 0.7	0.9 / 1.2	1.5 / 2.1
IDR	-2.4 / -1.5	-2.4 / -1.8	**-2.9 / -1.7**	-0.5 / -0.4	0.3 / 0.3	0.9 / 1.1	1 / 1.1	0.6 / 0.8	0.4 / 0.6	0.5 / 0.2	-0.7 / -0.3	-1.5 / -1.3
KUR	**-3.4 / -1.9**	**-3 / -1.8**	**-2.9 / -1**	**-0.6 / 0.1**	0.3 / 0.6	**0.7 / 1.2**	**0.8 / 1.2**	**0.7 / 1.1**	**0.4 / 1.1**	0.4 / 0.5	-1.1 / -0.4	**-2.7 / -1.6**
KAR	-1.1 / -0.2	-0.8 / 0.1	**-1.1 / 0.5**	0.5 / 0.4	0.8 / 0.6	0.5 / 0.9	0.9 / 1	0.9 / 0.9	0.5 / 0.9	0.8 / 0.8	-0.4 / 0.4	-1.1 / -0.3
ERM	**-1.1 / -0.1**	**-0.8 / 0.6**	**-0.2 / 1**	0.6 / 0.6	0.6 / 0.9	**0.8 / 1.1**	1 / 1.2	0.9 / 1.1	0.8 / 0.9	0.8 / 0.7	0.2 / 0.6	-0.5 / -0.1
MIN	**-2.7 / -0.8**	**-2.5 / -0.9**	**-1.3 / 0.2**	0.9 / 0.8	1.4 / 1.2	2.1 / 1.9	**2.4 / 2.1**	**2.1 / 1.7**	1.2 / 1.2	0.5 / 0.6	**-0.7 / 0.2**	**-1.9 / -0.5**
CHER	**2.4 / 6.3**	**2.1 / 5.7**	**1.1 / 3.7**	1.1 / 1.4	**1.3 / 0.8**	**1.1 / 0.6**	**1.1 / 0.9**	1.2 / 1.4	**1.3 / 2.2**	**1.8 / 3.0**	**2.0 / 4.5**	**2.2 / 6.1**
	Precipitation, $dP = P_{station} - P_{TASH}$ (mm)											
SHIRA	**-4 / -7**	-3 / -5	-2 / -3	-10 / -19	-22 / -29	-15 / -29	-7 / -17	**-1 / -21**	**-4 / -21**	-17 / -15	-11 / -13	**-5 / -10**
BOGR	-1 / -3	-2 / -5	**-3 / 2**	-15 / -6	-24 / -17	-20 / -20	-11 / -5	9 / -11	-5 / -16	-13 / -4	-9 / -15	**-2 / -8**
UYB	**-4 / -8**	**-3 / -6**	-4 / -6	-19 / -23	-28 / -37	**-14 / -32**	-17 / -18	-13 / -26	**-15 / -27**	-21 / -23	-13 / -17	**-6 / -11**
BEYA	not calculated											
IDR	8 / 2	2 / 2	-1 / -1	-6 / -16	-12 / -22	-24 / -29	-6 / -16	2 / -16	**9 / -7**	-1 / -4	1 / -3	7 / 4
KUR	8 / 5	6 / 6	3 / 1	-7 / -9	-11 / -15	-14 / -27	-16 / -14	4 / -12	8 / -3	1 / 5	6 / 7	13 / 11
KAR	11 / 8	6 / 6	5 / 5	-2 / -5	-5 / -10	-12 / -13	3 / -8	8 / 4	**19 / 4**	9 / 4	11 / 6	14 / 8
ERM	11 / 12	**12 / 7**	**12 / 7**	7 / 5	6 / 0	-2 / -5	3 / 3	13 / 3	**23 / 10**	7 / 15	14 / 11	17 / 12
MIN	-1 / -2	0 / -2	**1 / -3**	-13 / -15	-18 / -22	-20 / -21	-24 / -16	-14 / -14	1 / -7	-12 / -5	-6 / -9	**1 / -4**
CHER	3 / 0	4 / 0	4 / 2	1 / 1	4 / 5	8 / 4	7 / 19	33 / 14	**14 / 3**	**-2 / 11**	0 / 3	2 / 2
